# RNA-seq analysis of chlorogenic acid intervention in duck embryo fibroblasts infected with duck plague virus

**DOI:** 10.1186/s12985-024-02312-2

**Published:** 2024-03-07

**Authors:** Yunyun Yang, Qiandong Zhang, Haiqing Cai, Yi Feng, Anlin Wen, Ying Yang, Ming Wen

**Affiliations:** 1 School of Animal Science, Guizhou University, Guiyang, China; 2 Guizhou Provincial Animal Biological Products Engineering Technology Research Center, Guiyang, China

**Keywords:** Duck plague virus, Chlorogenic acid, Duck embryo fibroblasts, Transcriptomics, Histopathology

## Abstract

**Introduction:**

Chlorogenic acid, the primary active component in Chinese medicines like honeysuckle, exhibits anti-inflammatory and antiviral effects. It has been demonstrated that chlorogenic acid effectively prevents and treats Duck enteritis virus (DEV) infection. This study aims to further elucidate the mechanism by which chlorogenic acid prevents DEV infection.

**Methods:**

Duck embryo fibroblast (DEF) cells were pre-treated with chlorogenic acid before being infected with DEV. Cell samples were collected at different time points for transcriptomic sequencing, while qPCR was used to detect the proliferation of DEV. Additionally, 30-day-old ducks were treated with chlorogenic acid, and their lymphoid organs were harvested for histopathological sections to observe pathological damage. The proliferation of DEV in the lymphoid organs was also detected using qPCR Based on the transcriptomic sequencing results, NF-κB1 gene was silenced by RNAi technology to analyze the effect of NF-κB1 gene on DEV proliferation.

**Results:**

Compared to the viral infection group, DEF cells in the chlorogenic acid intervention group exhibited significantly reduced DEV load (*P* < 0.05). Transcriptomic sequencing results suggested that chlorogenic acid inhibited DEV proliferation in DEF cells by regulating NF-κB signaling pathway. The results of RNAi silencing suggested that in the three treatment groups, compared with the DEV experimental group, there was no significant difference in the effect of pre-transfection after transfection on DEV proliferation, while both the pre-transfection after transfection and the simultaneous transfection group showed significant inhibition on DEV proliferation Furthermore, compared to the virus infection group, ducks in the chlorogenic acid intervention group showed significantly decreased DEV load in their lymphoid organs (*P* < 0.05), along with alleviated pathological damage such as nuclear pyretosis and nuclear fragmentation.

**Conclusions:**

Chlorogenic acid effectively inhibits DEV proliferation in DEF and duck lymphatic organs, mitigates viral-induced pathological damage, and provides a theoretical basis for screening targeted drugs against DEV.

**Supplementary Information:**

The online version contains supplementary material available at 10.1186/s12985-024-02312-2.

## Introduction

Duck plague virus, also known as duck enteritis virus, is a member of the α-herpesvirus subfamily of the Herpesviridae family. It infects ducks, geese and other geese-like birds through the digestive tract and other ways, causing acute febrile septic infectious diseases characterized by extensive hemorrhage in the digestive tract and progressive degeneration of parenchymal organs, and is one of the most important pathogens that threaten and jeopardize the development of the waterfowl aquaculture industry [1, 2]. It has been found that after DPV infection enters the duck organism, it mainly attacks B lymphocytes, causing atrophy of the duck lymphoid organs and immunosuppression, which leads to secondary infection of other pathogens and accelerates the death of ducks [3–5]. At present, the prevention and control of DPV is mainly based on vaccination, but due to various reasons, sporadic disease in immunized animals may affect the healthy development of poultry farming, so there is an urgent need for drugs with unique efficacy for clinical prevention and treatment [6, 7].

Chlorogenic acid has antiviral activity against a variety of viruses, including hepatitis B virus, influenza virus, herpes simplex virus, adenovirus, HIV, coronavirus, porcine reproductive and respiratory syndrome virus, etc. Gui-Feng Wang et al. [8] evaluated the effect of chlorogenic acid on the activity of hepatitis B virus in a duck model by utilizing hepatitis B virus-infected ducks and HepG 2.2.15 cells as in vitro and ex vivo models. Using ducks and HepG2.2.15 cells infected with hepatitis B virus as an ex vivo model, they evaluated the effect of chlorogenic acid on the activity of hepatitis B virus, and the results showed that chlorogenic acid reduced the level of hepatitis B virus in serum, inhibited the replication of hepatitis B virus DNA and the production of viral surface antigens in the duck model. The study showed that chlorogenic acid could regulate the TGF-β1/SMAD3 pathway to attenuate hepatic fibrosis, while the levels of IL-6, TNF-α and LN were significantly reduced, i.e., chlorogenic acid could attenuate the degree of hepatic injury and the degree of inflammatory cell infiltration. Yinju Li et al. [9] treated chicken embryos inoculated with a potent strain of infectious bursal infection with chlorogenic acid, and the results suggested that there was a concentration-dependent response in the antiviral activity of chlorogenic acid in duck models. There was a concentration-dependent response of antiviral activity, inhibition of NF-κB activation and expression levels of pro-inflammatory cytokines TNF-α, IL-1β and histamine. In the study of Xiang Li et al. [10], chlorogenic acid was able to exert antiviral function by interfering with transcription and translation at the early stage of enterovirus replication and inhibiting the secretion of IL-6, TNF-α, IFN-γ and MCP-1. In summary, the anti-inflammatory and anti-viral functions of chlorogenic acid are mostly related to the direct inhibition of NF-κB activation or the indirect effect of upstream signaling pathways on NF-κB signaling and the inhibition of the expression of a variety of pro-inflammatory factors. Therefore, the study of chlorogenic acid can help prevent and treat viral infections and other related diseases.

By using honeysuckle powder in the treatment of ducklings artificially infected with DPV, Zhang et al. [11] found that honeysuckle could improve the anatomical symptoms, pathological and histological changes, and immune organ function of DPV-infected ducks, but the molecular mechanism of its action has not yet been analyzed. Therefore, this study intends to select chlorogenic acid, an antiviral active ingredient of honeysuckle, as the research object on the basis of the previous study, and investigate the molecular mechanism of chlorogenic acid to inhibit the proliferation of DPV by using qRT-PCR and transcriptome sequencing technology, with a view to providing scientific basis for the prevention and treatment of DPV by the medicine.

## Materials and methods

### Virus strain, animals and recombinant plasmid

The Guizhou strain of duck plague virus (DPV-GZ strain, TCID50 3.16 × 10^–9^/0.1 mL) was provided by Guizhou Animal Bioproducts Engineering and Technology Research Center; 30-day-old San Sui ducks, which were tested to be free of DPV and its antibodies, were provided by San Sui Duck Breeding Farm of Guizhou Province. pGPU6/GFP/Neo-NF-κB was provided by Animal Disease Research Laboratory of Guizhou Province.

### Preparation of duck embryo fibroblasts

SPF-fertilized duck eggs were incubated for 11 d. The embryos were taken out, and the head, limbs and viscera were removed with ophthalmic scissors; the remaining tissues were washed with PBS and cut into pieces, and then introduced into the appropriate amount of trypsin, which was transferred to 10 ml centrifuge tubes by pasteur pipette, and then put in a water bath for digestion at 37℃ for 20 min, and then inverted and mixing once every 5 min. After digestion, centrifuge at 3000 rpm for 5 min, pour off the trypsin in an ultra-clean bench, add 10% fetal bovine serum medium to terminate the digestion, and mix upside down. The filtrate was filtered through a 70 μm pore size cell sieve, and the filtrate was duck embryo fibroblasts.

### Chemicals and reagents

Chemicals: Chlorogenic acid powder was purchased from Mingze Biotechnology Co. Chlorogenic acid capsules were prepared by dispensing chlorogenic acid powder into edible grade enteric melt capsule shells.

Reagents: Prime ScriptTM RT Master Mix was purchased from TaKaRa; Cell Counting Kit-8 was purchased from Tongren Institute of Chemical Research; methanol and acetonitrile were purchased from Thermo Fisher Scientific; ammonia formate was purchased from Honeywell Fluka, formic acid from DIMKA; xylene, anhydrous ethanol and light liquid paraffin were purchased from Sinopharm Chemical Reagent Co. Ltd; xylene, anhydrous ethanol and light liquid paraffin were purchased from Sinopharm Chemical Reagent Co. 4% paraformaldehyde solution and HE dye solution were purchased from Xavier Biotechnology Co.

### Determination of chlorogenic acid concentration

DEF cells were inoculated into 96-well culture dishes, and different concentrations of chlorogenic acid (final concentrations of 0, 0.125, 0.25, 0.5, 1, 2 and 4 mg/mL) of 100 μL were configured with the maintenance medium (containing 3% fetal bovine serum) as solvent, respectively, and three replicate wells were set up for each concentration, and the cells were cultured for 24 h. The medium was aspirated and discarded from the wells, and the maintenance medium containing 10% CCK-8 solution was added. After incubation for 24 h, the medium in the wells was aspirated, and the OD value at 480 nm was detected by an enzyme labeler after 1 h of incubation.

### Experimental groups and treatments

**Fig. 1:**
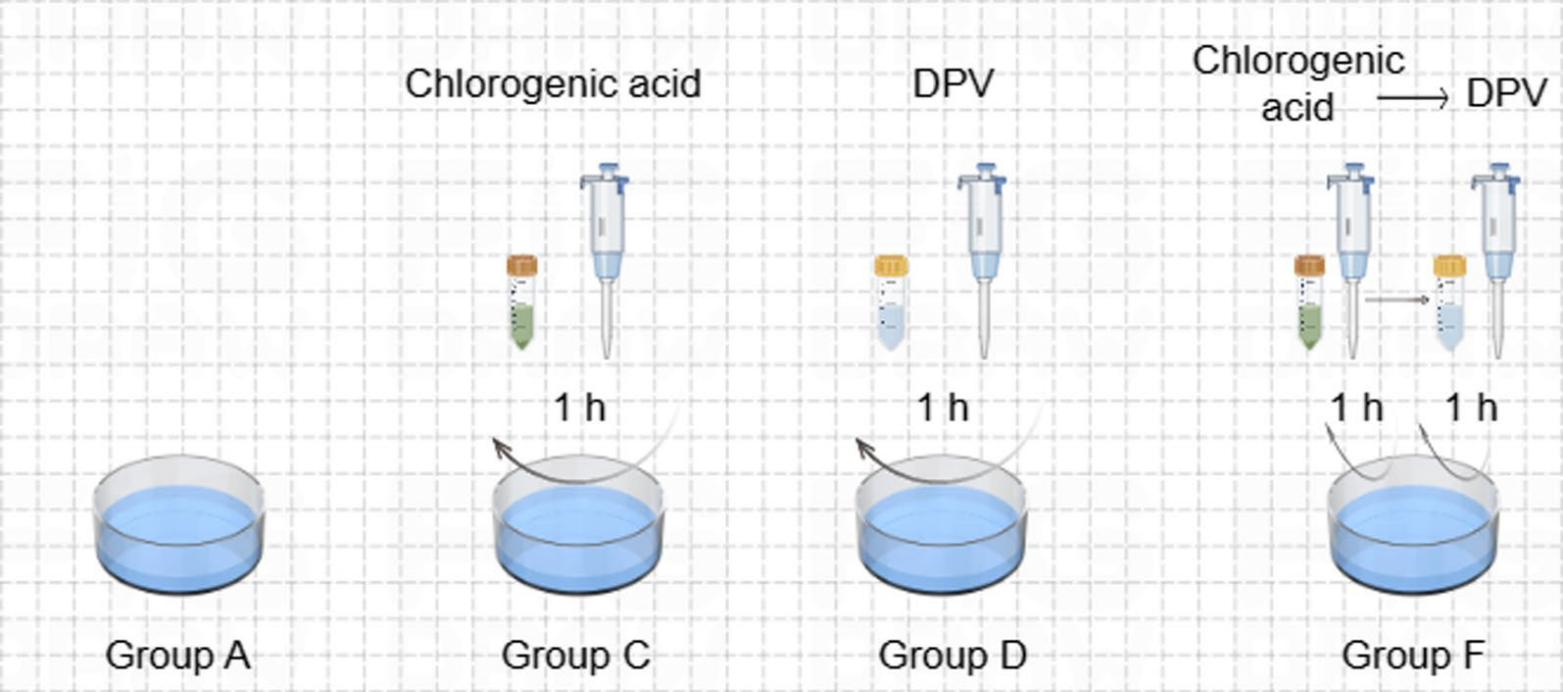
Experimental grouping and treatment. Group A. After the cell adherent grew to 70%, the maintenance medium was changed and continued for 24 h, 36 h and 48 h. Group C. 0.250 mg·mL-1 chlorogenic acid was prepared with maintenance medium as solvent, 100 μL was added to each well for 1 h and aspirated, Hanks solution was rinsed twice, and 100 μL of maintenance medium was added to continue to culture for 24 h, 36 h and 48 h. Group D. 100 μL of DPV virus solution diluted in maintenance medium was added for DEF 1 h and aspirated. Hanks was rinsed twice and then added to the maintenance medium for 24 h, 36 h and 48 h. Group F. DEF was aspirated with 0.250 mg·mL-1 chlorogenic acid for 1 h, Hanks was rinsed twice and inoculated with DPV for 1 h, and Hanks was rinsed twice and then the maintenance medium was added for 24 h, 36 h and 48 h

The cells were grouped and processed according to Fig. 1, and the cell samples were collected for 24 h, 36 h and 48 h, rinsed with Hanks solution for 3 times, centrifuged at 3000r/min for 10 min, discarded with Hanks solution, and transferred to a − 80 °C freezer for 15 min and then transferred to a − 80 °C freezer for later use.

### Virus copy number was detected by qRT-PCR and transcriptomic analysis was performed

Cells were inoculated into 96-well plates, and after the cells were wall-adhered to the wall as in Table 1, they were randomly grouped and treated, and the cellular activity of DEF in each group at 24 h was determined by CCK-8 method. In Table 1, total RNA was extracted by Trizol method according to the instructions, and was reverse-transcribed into cDNA using TaKaRa reverse transcription kit. qRT-PCR was performed using cDNA as a template, and the information of the qRT-PCR primers is shown in Additional file 1: Table S1. qRT-PCR was performed according to the instruction manual of TB Green Premix Ex Taq II. The total volume of the reaction was 20 μL, and the reaction program was 95 °C for 30 s, 95 °C for 5 s and 60 °C for 34 s for 40 cycles, and the amplification results were calculated by the 2^−ΔΔct^ method.

**Table 1:** Determination of safe drug concentrations

	N	0	0.125	0.25	0.5	1	2	4
Chlorogenic acid	0.158	0.701	0.694	0.691	0.697	0.181	0.213	0.205
	0.154	0.727	0.671	0.699	0.696	0.244	0.194	0.186
	0.146	0.711	0.705	0.715	0.692	0.272	0.176	0.16

The cell samples stored in 2.4. Medium-80 °C refrigerator were packed in dry ice and sent to Shenzhen BGI Co., Ltd. for transcriptomic sequencing using DNB platform sequencer (MGI2000). And sequencing method can be reference for building a concrete link: https://www.yuque.com/yangyulan-ayaeq/oupzan/kn1ley7iz2c8ylgr

### Validation of differential genes

To verify the reliability of the transcriptomics sequencing results of chlorogenic acid inhibition of DPV proliferation in DEF cells, eight differential genes were selected and designed according to the gene sequences of GenBank for TNFAIP2, IFN-AR1, TNFSF15, CCL26, CHCHD10, ROR2, IL-16, and GNG10, respectively; the cells in the Table 1 Sample cDNA samples were amplified by qRT-PCR using Additional file 1: Table S1 primers, and the amplification program was 95 °C for 30 s, 95 °C for 5 s and 60 °C for 34 s for 40 cycles. The relative expression of mRNA of differentially expressed genes was calculated by the 2^−ΔΔct^ method using β-Actin as the internal reference gene and compared with the transcriptional sequencing results.

### Effect of targeting NF-kB1 gene RNAi on DEV proliferation

The cells were inoculated with six-well plates and randomized and treated according to the following:

The first group was transfected with pGPU6/GFP/Neo-NF-κB1 24 h after DEV infection.

The second group was infected with DEV after transfection of pGPU6/GFP/Neo-NF-κB1 for 24 h.

The third group was simultaneously infected with DEV and transfected with pGPU6/GFP/Neo-NF-κB1.

At the same time, each group was set up three replicates (DEV + interference recombinant plasmid), DEV group (only added DEV), negative group (only added interference recombinant plasmid) and blank group (only DEF cells). The experimental group was interference plus virus group, DEV group was virus group, negative group was interference group, and blank group was cell group. Cell products were collected at 24, 36 and 48 72 h after culture, total RNA was extracted and reverse-transcribed into cDNA. The content of DEV-NP gene was detected by QRT-PCR as above.

### Animal experimentation

Animal breeding, use, and sample collection were performed according to the guidelines of the Laboratory Animal Center of Guizhou University, and all animal procedures were approved by the Ethics Committee and the Laboratory Animal Care and Use Committee (Permit Number: EAE-GZu-2020-E018, 2 September 2020) of Guizhou University (Guizhou, China). Thirty 30-day-old mallard ducklings were randomly divided into three groups, i.e., normal control group (N), virus-infected group (V) and chlorogenic acid intervention group (M), with 10 ducks/group. ducks in group N were injected intramuscularly with sterilized saline (0.2 mL/each); ducks in group V were injected intramuscularly with DPV (0.2 mL/each); and ducks in group M were injected intramuscularly with DPV (0.2 mL/each), and at the same time, they were manually administered with 1 chlorogenic acid capsule (containing 0.6 ± 0.6% of chlorogenic acid). Group M ducks were injected with DPV (0.2 mL/each duck) and manually administered 1 capsule of chlorogenic acid (0.6 ± 0.02 g/capsule), 1 capsule per day until the end of the 5-d test. The ducks were kept in isolation, and the clinical symptoms were observed every day. The ducks were euthanized at 24 h, 36 h and 48 h after virus infection, and the spleen, thymus and bursa tissues of the ducks in each group were collected aseptically, fixed in paraformaldehyde solution, dehydrated, embedded and then prepared into pathology sections and stained by HE, and the pathology of the immune tissues and organs of the ducks in each group was observed under the microscope; the viral DNA of the lymphoid organs of the ducks in each group was extracted from each group at each time point as templates, and the viral DNA was used for the templates. According to the manufacturer's instructions, the viral DNA of each group of ducks collected at each time point was extracted as a template, and the viral load of DPV was detected by qPCR, and the sequences of DPV primers are shown in Additional file 1: Table S1.

### Statistical analyses

The experimental data of SYBR green-based real-time PCR were expressed as mean ± standard deviation (${\overline{\text{X}}} \pm {\text{S}}$). SPSS 26.0, CaseViewer, prism and other software were used for analysis and mapping. *: 0.01 < *P* < 0.05 indicated significant differences. **: *P* < 0.01, the difference is very significant.

## Results

### Determination of drug concentration

In order to eliminate the toxic effect of chlorogenic acid on DEF and control the effect of chlorogenic acid on DEF within the safe range, the optimal safe concentration of the drug was determined. As shown in Table 1, with the increase of concentration, the cell activity showed the trend of increasing and then decreasing, but all of them were lower than the cell activity of 0 mg/mL, and the degree of decrease of the cell activity was not significant, which indicated that the low concentration of chlorogenic acid had a small toxicity on DEF, and the cell activity was the highest when the concentration was up to 0.250 mg/mL, which was selected as the safe concentration of the test drug.

Group N, to which only 3% maintenance medium + CCK-8 solution was added, is the solution control group, and the concentration of 0 mg/mL without chlorogenic acid was added, and is the cell control group.

### Cell activity assay

In order to understand whether chlorogenic acid will alleviate the effect of DPV on DEF cells when acting on infected cells, the CCK-8 method was used to detect the cell activity of each group of cells after different treatments at 24 h, 36 h and 48 h of incubation, as shown in Table 2, the cell activity of group C did not change significantly compared with group A (*P* > 0.05), and the cell activity of group F increased extremely significantly compared with group D (*P* < 0.01). It indicates that DPV infection leads to a decrease in cell activity, and the cell activity of DEF cells after chlorogenic acid action is significantly increased compared to no treatment in DPV infection.

**Table 2:** Results of DEF cell viability assay in each group

	24 h	36 h	48 h
Blank control group (A)	0.78 ± 0.058^Aa^	0.73 ± 0.021^Aa^	0.86 ± 0.031^Aa^
Chlorogenic acid control group (C)	0.72 ± 0.025^Aab^	0.70 ± 0.047^ABa^	0.74 ± 0.045^ABab^
DPV control group (D)	0.57 ± 0.067^Bc^	0.49 ± 0.056^Cc^	0.36 ± 0.127^Cc^
Chlorogenic acid intervention group (F)	0.68 ± 0.03^ABb^	0.61 ± 0.031^Bb^	0.65 ± 0.015^Bb^

Inconsistencies in lowercase letters a, b, c in the table indicate significant differences, and inconsistencies in uppercase letters A, B, C indicate significant differences

Group A was the cell control group, group C was the chlorogenic acid control group, group D was the virus control group, and group F was the chlorogenic acid intervention group

### Infected cell virus qRT-PCR detection results

**Fig. 2:**
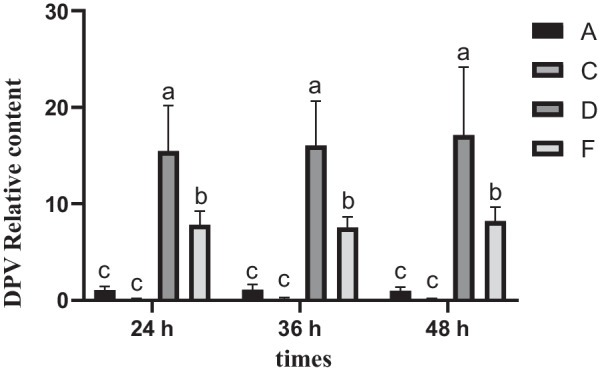
Detection of viral load at various periods after the action of chlorogenic acid on infected cells the same lowercase letters represent non-significant differences between the groups compared at the same time, and different lowercase letters represent significant differences between the groups compared at the same time, followed by the same

In order to understand the viral load of cells in each group at each time point after DPV infection, qRT-PCR was used. As shown in Fig. 2, compared with the cell control group, the DPV load of the virus-infected group increased significantly at all three time points (*P* < 0.05); compared with the virus-infected group, the DPV load of the cells in the chlorogenic acid-intervention group showed a significant decrease at all three time points (*P* < 0.05), which indicated that chlorogenic acid could inhibit the proliferation of DPV in DEF cells.

### Transcriptomics analysis results

#### Global changes in gene expression

**Fig. 3:**
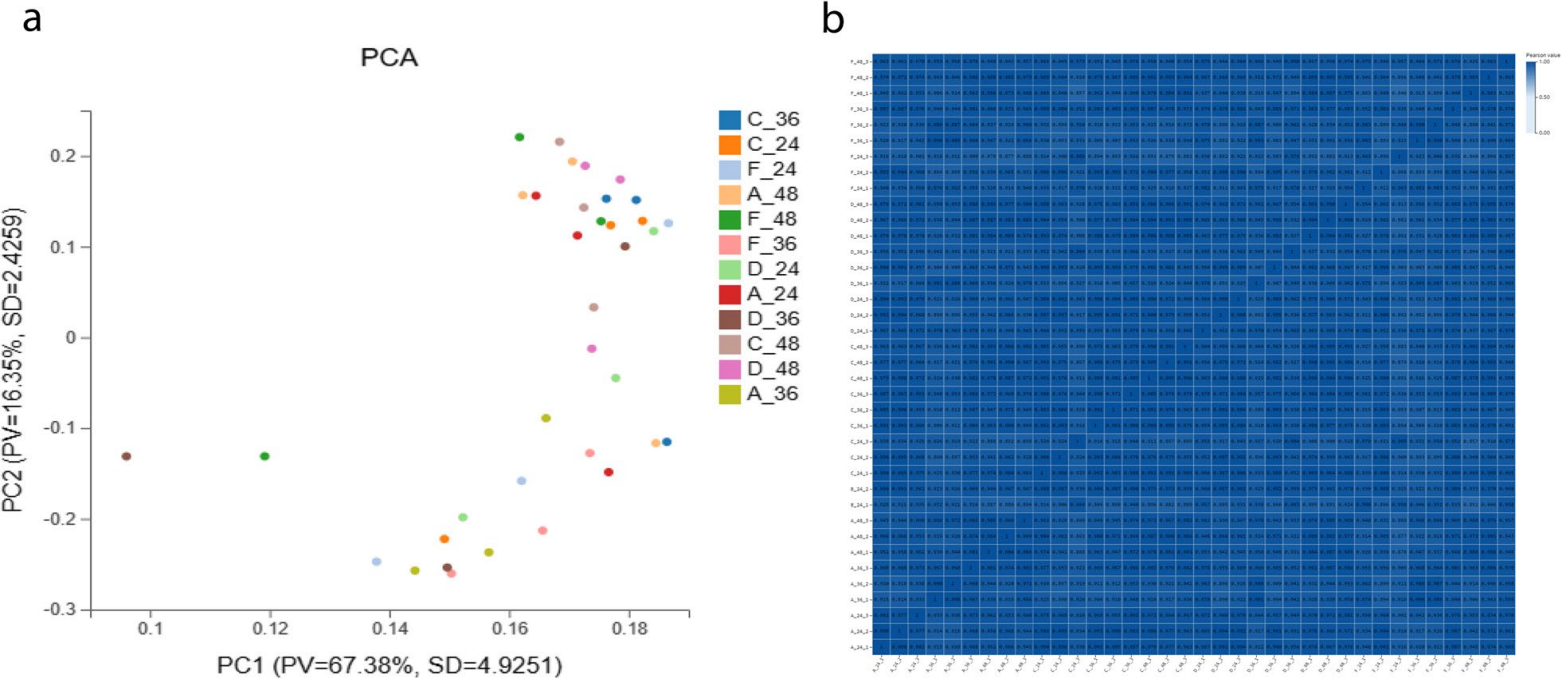
Sample relationship analysis. **a** sample principal component analysis plot, PCA1 is the first principal component, PCA2 is the second principal component, and percentage refers to the contribution of the comparison sample; **b** sample correlation heat map

**Fig. 4:**
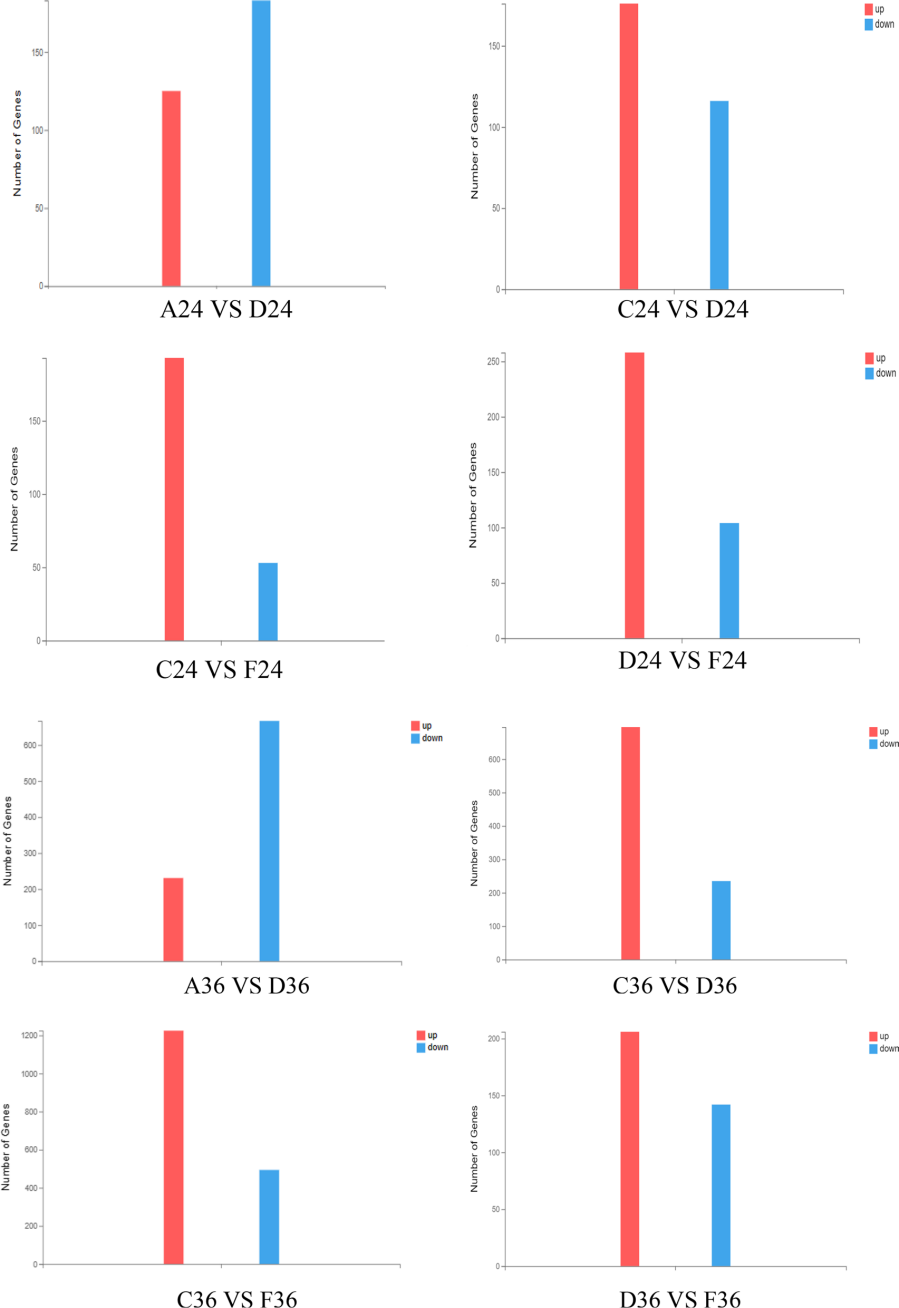

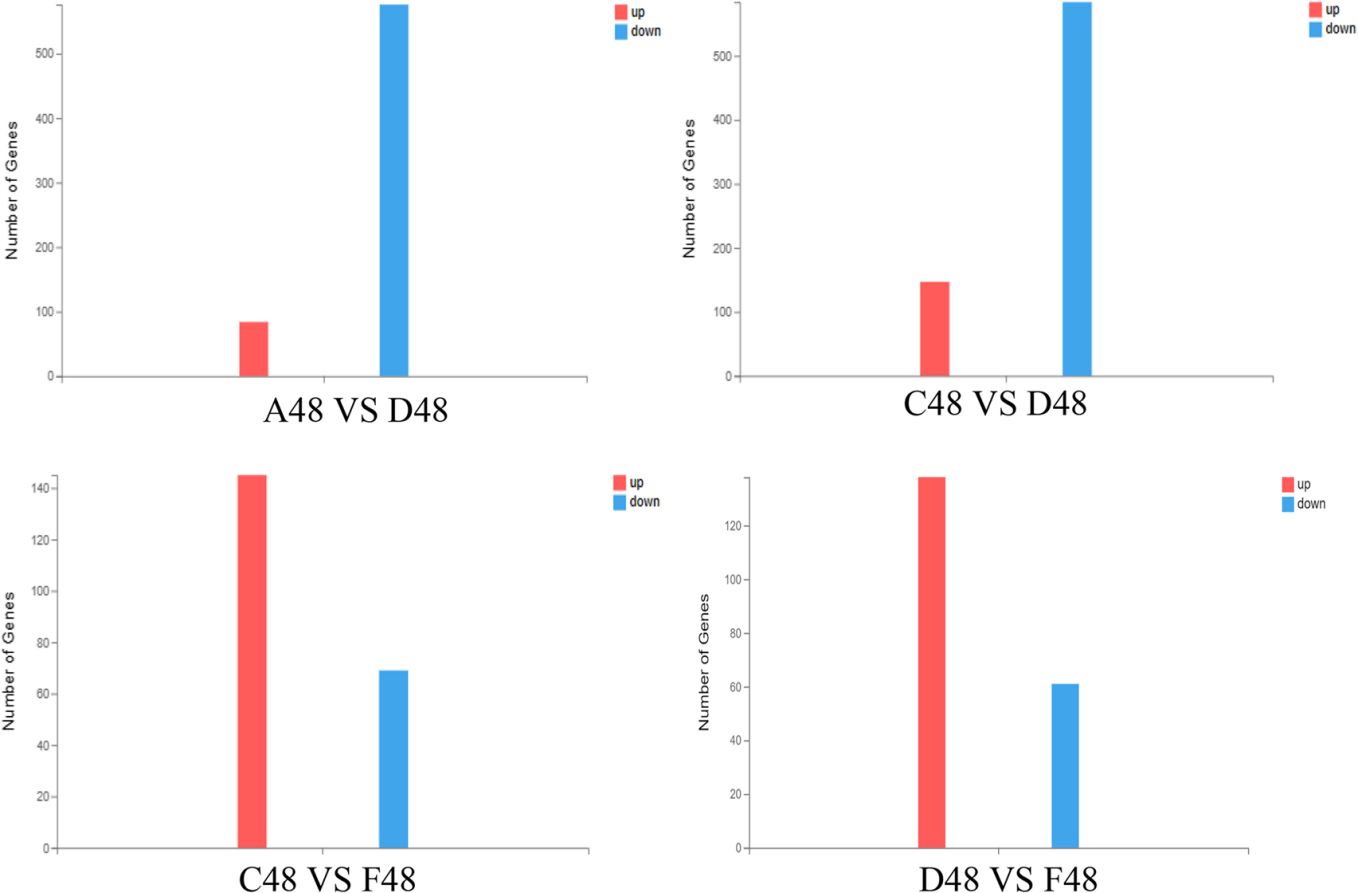
Overall statistical results of differential genes. The X axis represents the difference comparison scheme for each group, and the Y axis represents the corresponding number of differential genes (DEG). Red represents the number of up-regulated DEGs and blue represents the number of down-regulated DEGs

In order to study the gene expression of chlorogenic acid during DPV infection of DEF, total cellular RNA was extracted, and sequencing library was constructed and used for RNA-seq. To reduce the interference of subsequent sequencing, the data were filtered and clean reads were obtained, and the percentage of clean reads of high-quality data obtained after filtration was higher than 90%, and the overall quality of the data was high, which lays the foundation of subsequent analyses (Additional file 2: Table S2). The results of PCA showed that the distribution of samples in the same group was more concentrated, and the distribution between samples in different groups was more dispersed, suggesting that the samples were biologically well duplicated (Fig. 4a); the heat map of sample correlation showed that the samples in the same group were similar in color, and the correlation was high (Fig. 4b). Overall statistical analysis of differential genes and visualization by volcano diagram (Additional file 3: Fig. S1) showed that the number of down-regulated differential genes was significantly higher than the number of up-regulated differential genes in group A compared to group D, suggesting that DPV infection with DEF mainly caused down-regulation of gene transcript levels; the number of up-regulated differential genes was significantly higher than the number of down-regulated genes in group C compared to group F, and the number of up-regulated differential genes was significantly higher than the number of down-regulated genes in group D compared to group F, suggesting that chlorogenic acid mainly causes up-regulation of genes to inhibit DPV proliferation in DEF (Fig. 3).

#### Results of overall statistical analysis of differential genes

Comparison between groups at the same time point, the results are shown in Fig. 4: when chlorogenic acid and DPV acted DEF for 24 h, there were a total of 308 differentially expressed genes in group A and D, of which 125 were up-regulated genes and 183 were down-regulated expressed genes; a total of 292 differentially expressed genes in group C and D, of which 176 were up-regulated genes and 116 were down-regulated genes; a total of 246 differentially expressed genes in group C and F, of 193 up-regulated genes and 53 down-regulated genes; group D and group F differentially expressed genes totaled 362, including 258 up-regulated genes and 104 down-regulated genes.when chlorogenic acid and DPV acted DEF for 36 h, there were a total of 898 differentially expressed genes in group A and D, including 231 up-regulated genes and 667 down-regulated genes; there were a total of 931 differentially expressed genes in group C and D, including 696 up-regulated genes and 235 down-regulated genes; there were a total of 1719 differentially expressed genes in group C and F, including 1225 up-regulated genes and 494 down-regulated genes; and there were a total of 362 differentially expressed genes in group D and F. 494 down-regulated genes; Group D and Group F differentially expressed genes totaled 348, including 206 up-regulated genes and 142 down-regulated genes.when chlorogenic acid and DPV acted DEF for 48 h, the total number of differentially expressed genes in group A and D was 660, including 84 up-regulated genes and 576 down-regulated genes; the total number of differentially expressed genes in group C and D was 731, including 147 up-regulated genes and 584 down-regulated genes; the total number of differentially expressed genes in group C and F was 223, including 154 up-regulated genes and 69 down-regulated genes; the total number of differentially expressed genes in group D and F was 1425, including 206 up-regulated genes and 142 down-regulated genes. The total number of differentially expressed genes in group C and group F was 223, 154 up-regulated genes and 69 down-regulated genes; the total number of differentially expressed genes in group D and group F was 199, 138 up-regulated genes and 61 down-regulated genes.

The above results showed that the number of down-regulated genes was significantly higher than the number of up-regulated genes in group A compared with group D, suggesting that the infection of DEF by DPV mainly caused the down-regulation of gene transcription level; the number of up-regulated genes was significantly higher than the number of down-regulated genes in group C compared with group F, and the number of up-regulated genes was significantly higher than the number of down-regulated genes in group D compared with group F, suggesting that chlorogenic acid mainly caused gene up-regulation to inhibit the proliferation of DPV in DEF.

### Differential gene GO enrichment analysis

**Fig. 5:**
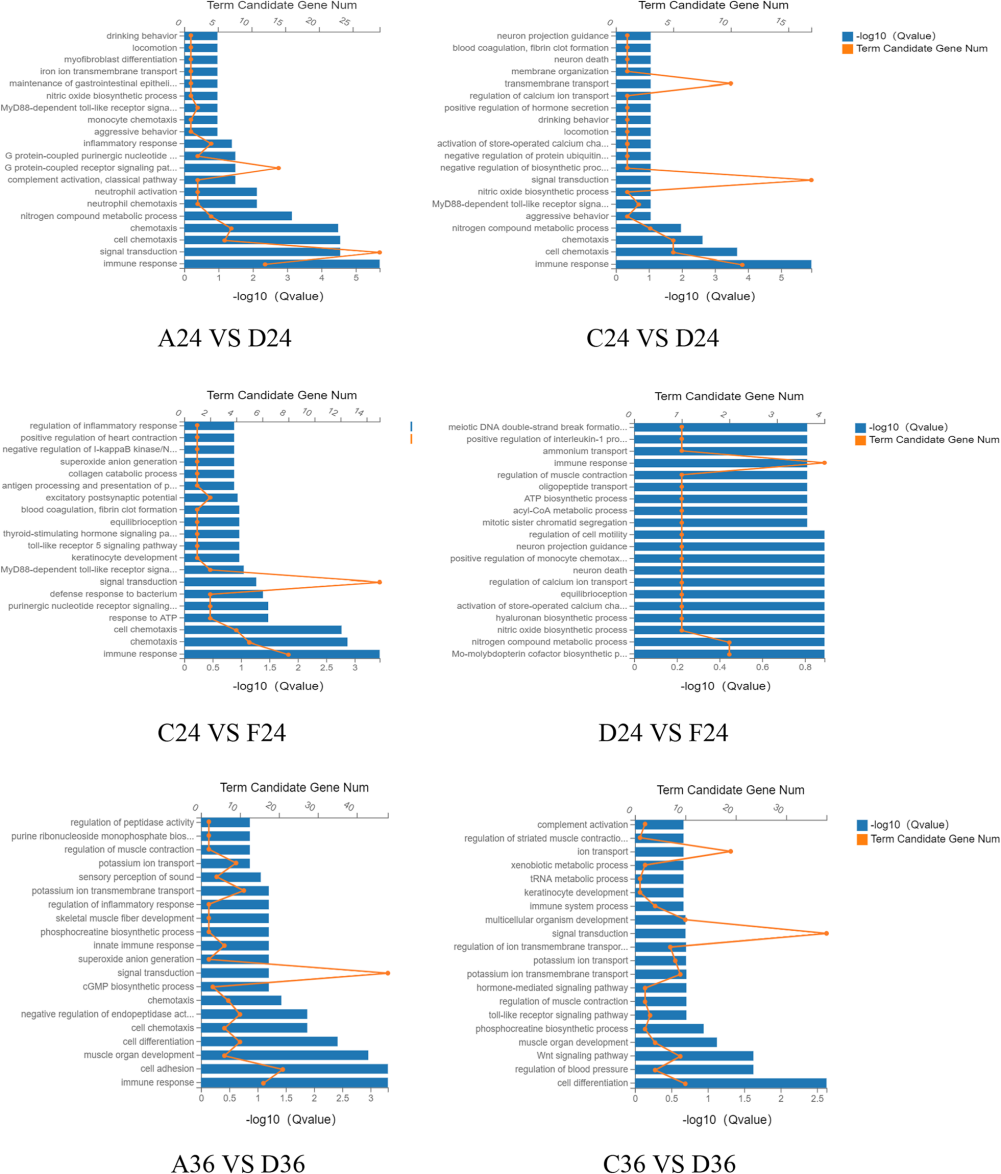

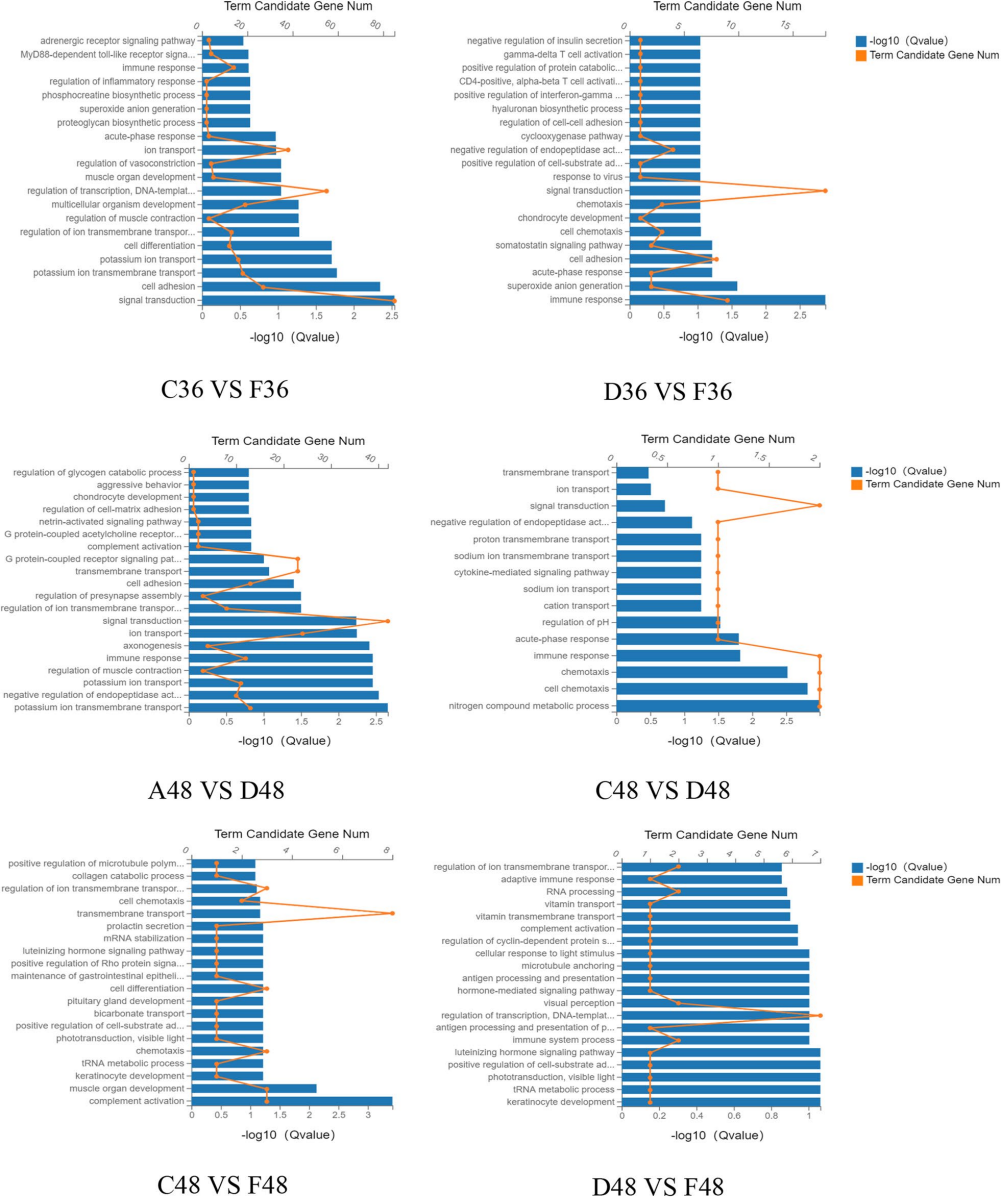
GO enrichment analysis of differentially expressed genes involved in biological processes. The X-axis is -lg (Qvalue), the Y-axis is the name of the participating function, and the broken line is the number of differential genes

The differentially expressed genes appeared in DPV-infected DEF cells after chlorogenic acid intervention were analyzed and categorized by comparing with GO database in order to observe which biological processes the differentially expressed genes were involved in. As shown in Fig. 5, the differential genes were mainly involved in biological processes such as immune response, cell adhesion, signal transduction, cell chemotaxis, and viral defense response at 24 h post-intervention; at 36 h post-intervention, the differential genes were mainly involved in biological processes such as immune response, inflammatory response, neutrophil chemotaxis, and activation of neutrophils; and at 48 h post-intervention, the differential genes were mainly involved in immune response, cell adhesion, activation of complement, cell chemotaxis, acute phase response, and cytokine-mediated signaling pathway.

### KEGG enrichment analysis of differentially expressed genes

**Fig. 6:**
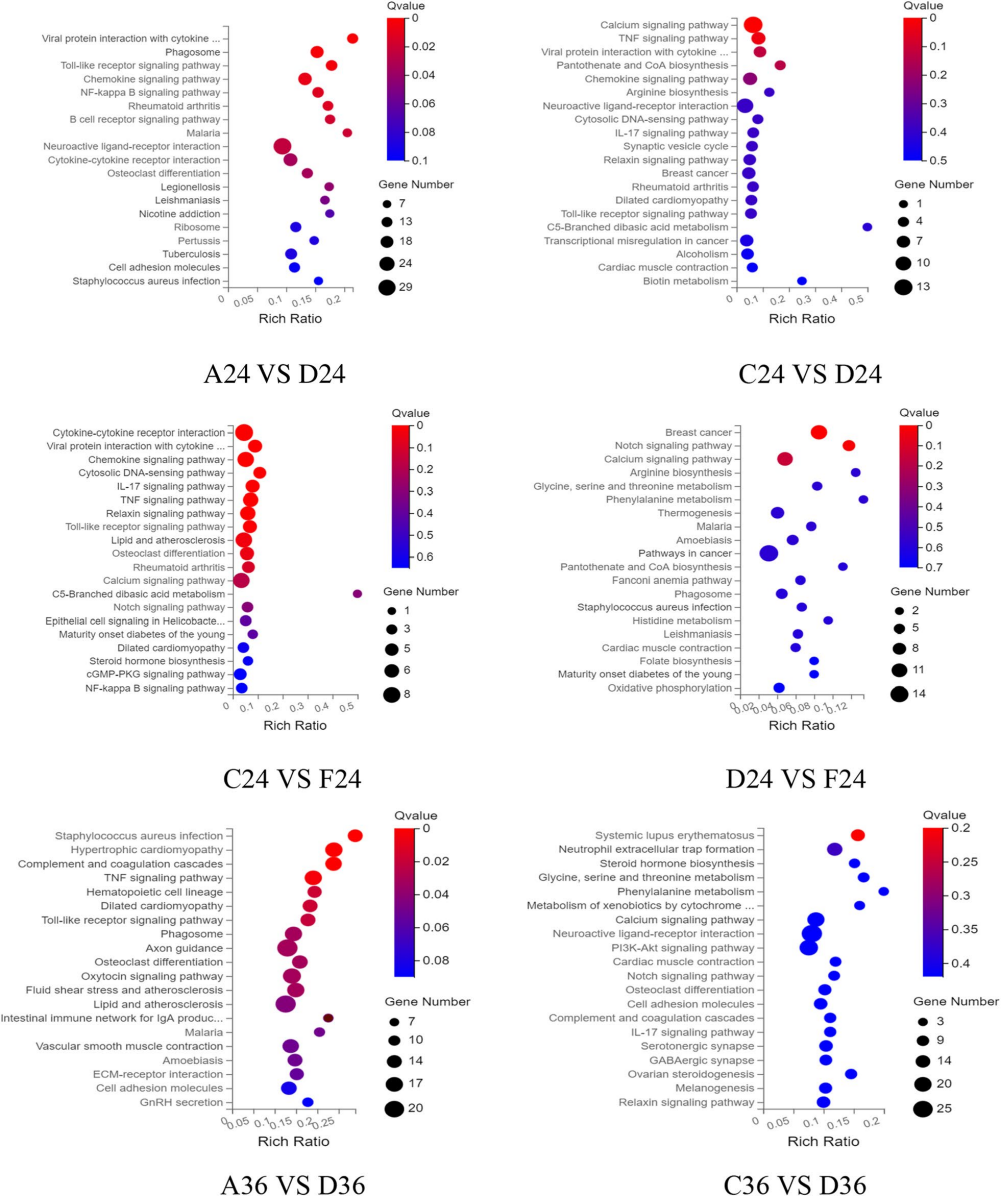

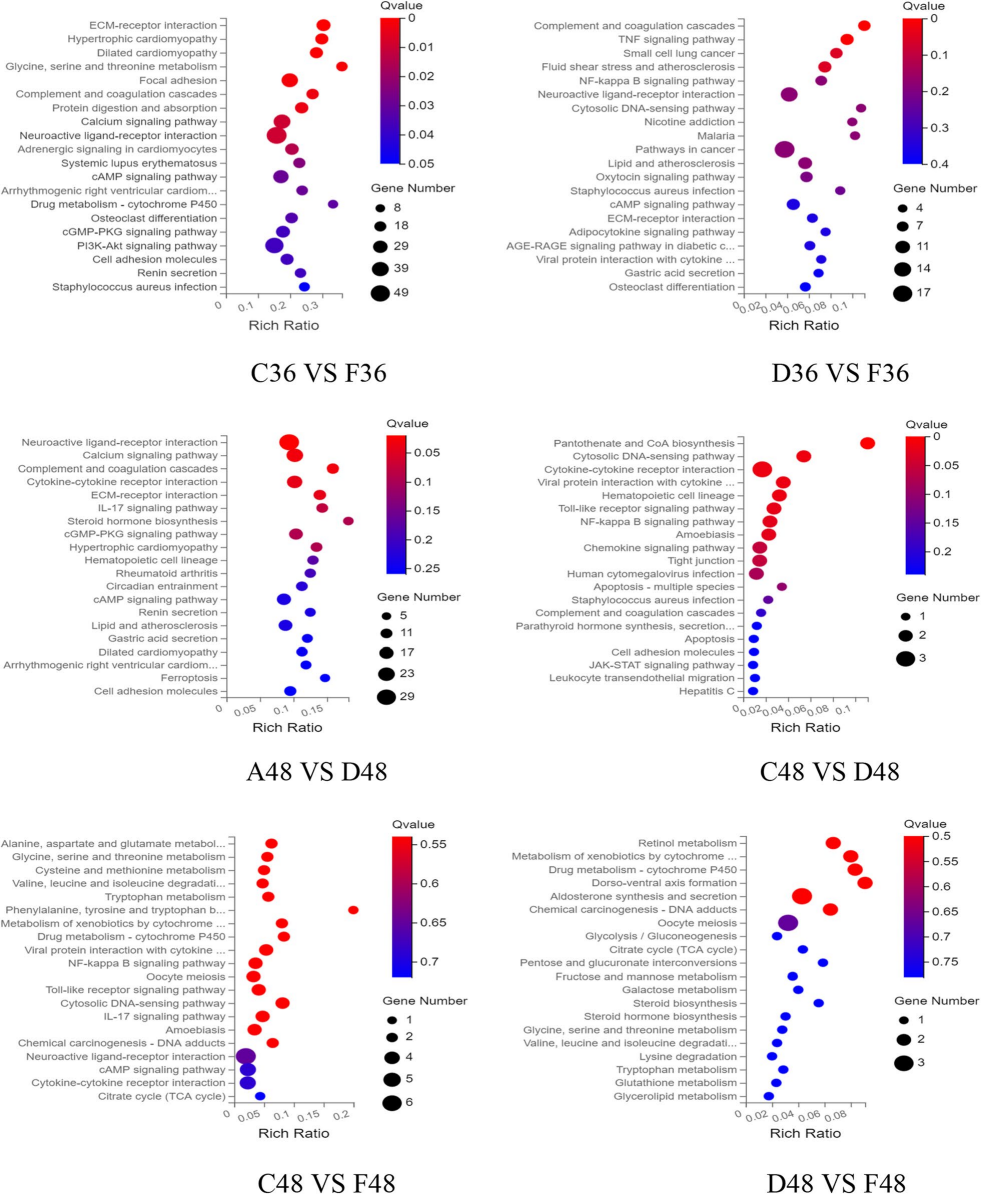
Bubble enrichment of KEGG pathway of differentially expressed genes. X axis is enrichment ratio, Y axis is KEGG Pathway, bubble size represents the number of genes annotated on a KEGG Pathway, color represents enrichment significance value, the redder the color, the smaller the significance value

The differential metabolites were compared with KEGG large database to analyze the enrichment of differential genes in relevant pathways after chlorogenic acid intervention in DPV-infected DEF cells. As shown in Fig. 6, the differentially expressed genes at 24 h of intervention were mainly involved in signaling pathways such as toll-like receptor signaling pathway, chemokine signaling pathway, NF-kappa B signaling pathway, TNF signaling pathway and IL-17 signaling pathway. The differentially expressed genes at 36 h mainly participated in TNF signaling pathway, NF-kappa B signaling pathway, calcium signaling pathway, toll-like receptor signaling pathway and DNA sensing pathway. The differentially expressed genes at 48 h were mainly involved in DNA sensing pathway, NF-kappa B signaling pathway, TNF signaling pathway and IL-17 signaling pathway.

### Correlation factor analysis and validation results

#### Data preprocessing and quality control

**Fig. 7:**
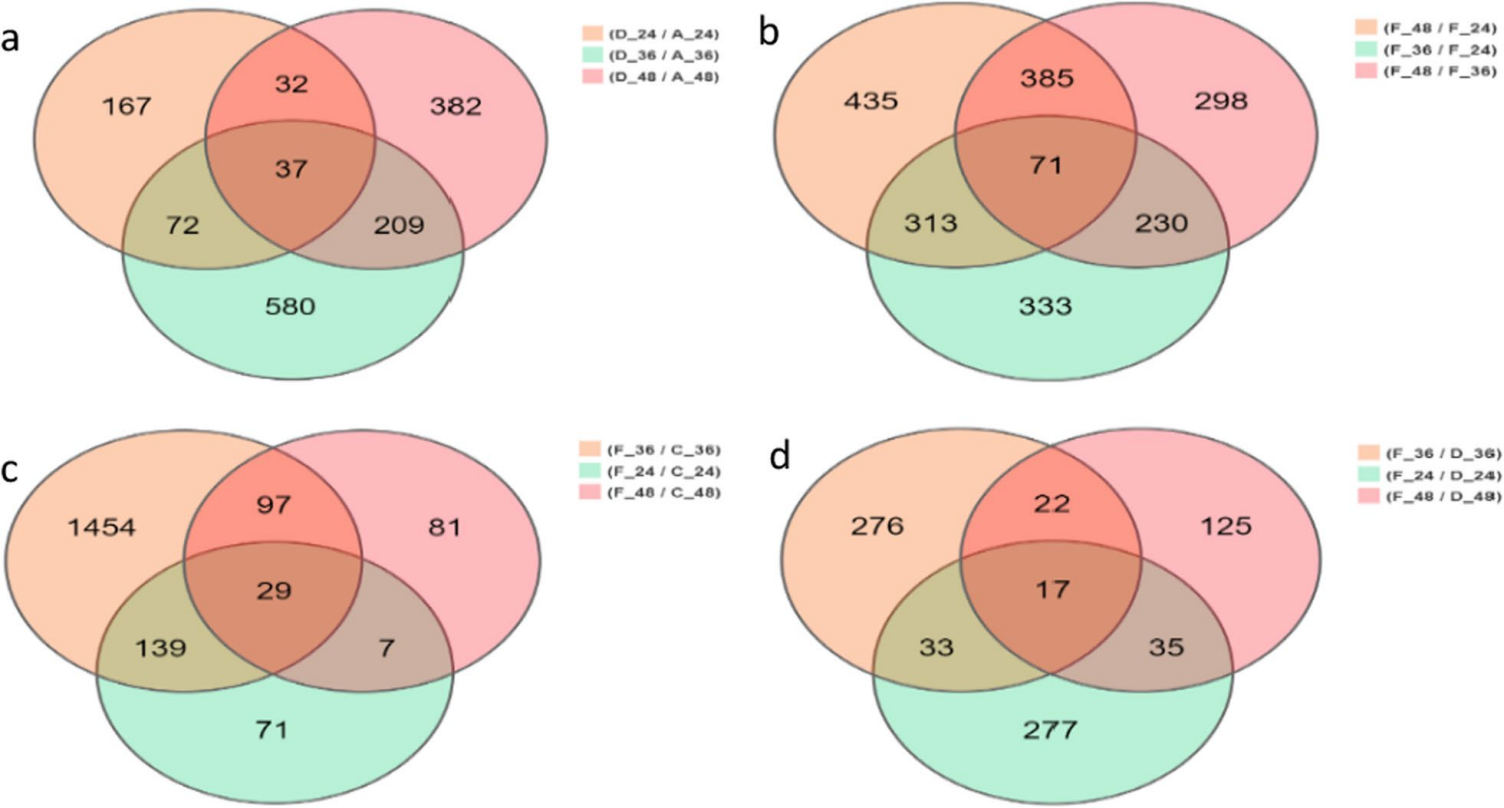
VENN analysis results of differentially expressed genes in each group. **a** Comparison at various time points between the virus group and the normal control group; **b** Each time point in the intervention group was compared respectively; **c** Comparison between intervention group and chlorogenic acid control group at each time point; **d** Comparison between intervention group and virus group at various time points

As shown in Fig. 7, the overlap in the VENN plot comparing the normal control group with the viral control group at each time point indicates 37 differentially expressed genes (Fig. 7a), the overlap in the VENN plot comparing the chlorogenic acid intervention group with each other at each time point indicates 71 differentially expressed genes (Fig. 7b), the overlap in the VENN plot comparing the chlorogenic acid intervention group with the chlorogenic acid control group at each time point indicates 29 differentially expressed genes (Fig. 7c), and the overlap in the VENN plot comparing the chlorogenic acid intervention group with the viral control group at each time point indicated 17 differentially expressed genes (Fig. 7d). Pooling these genes, the screening enumerated 8 meaningful differentially expressed genes, of which TNFSF15, TNFAIP2, IFNAR1 and CCL26 were pathway-related genes. RT-qPCR was used to analyze the above 8 DEGs to verify the accuracy of the RNA-seq data, and the results showed that the overall trend of the selected genes was consistent, proving the reliability of our RNA sequencing (Additional file 4: Fig. S2). In addition, the differentially expressed genes were annotated to the KEGG enrichment signaling pathway to analyze the expression changes of genes in the NF-κB dominated signaling pathway (Table 3).

**Table 3:** Analysis results of NF-κB, toll like receptor and TNF signal pathway

Channel name	Time period (h)	Adjust upwards and downwards	Gene
NF-κB signal pathway	24	↑	*MIP-1β*, *IL-8* and *IkBα*
		↓	*BTK*, *CARD11*, *PRCKB*, *PLCG2*, *BLNK*, *CD14*, *TLR4* and *TNFSF11*
	36	↑	*MIP-1β*, *IkBα*, *VACM-1*, *MD-2*
		↓	*COX2*
	48	↓	*TNFAIP3* and *COX2*
Toll like receptor signaling pathway	24	↑	*IL-8*, *MIP-1β*, *CCL26* and *IkBα*
		↓	*BTK*, *TLR2*, *TLR4*, *TLR5*, *CD14*, *TLR7/8*, *AP1* and *CD80*
	36	↓	*PI3K*, *TLR4*, *TRAF3*, *OPN*, *CD86*, *IkBα*, *MIP-1β*, *IL-6*, *RANTES* and *MD-2*
	48	↑	*IKBKE*
		↓	*MIP-1β*
TNF signaling pathway	24	↑	*CCL20*, *Mmp3*, *Mmp9*, *CCL26*, *VEGFD*, *IL18R1*, *MLKL* and *IkBα*
	36	↑	*CX3CL1*, *Mmp3*, *VCAM1*, *CD86* and *IkBα*
		↓	*LIF*, *SOCS3*, *EDN1* and *PTGS1*
	48	↓	*CREB5*, *CCL26*, *LIF*, *TNFAIP3*, *MMP9* and *PTGS2*

#### Results of critical path analysis

As shown in Table 4, chlorogenic acid inhibits DPV proliferation in vitro, and up- and down-regulates genes in the NF-κB signaling pathway, toll-like receptor signaling pathway, TNF signaling pathway, and IL-17 pathway at different time intervals. IL-6 and IL-8 are inflammatory factors, and IL-8 transmits signals to activate the toll-like receptor signaling pathway, and TLR4 and NFKBIA are NF-κB signaling inhibitors. pathway inhibitors, chlorogenic acid can regulate IL-8, TLR4 and NFKBIA, down-regulate the inflammatory-related factors in the toll-like receptor signaling pathway, coordinate IL-18R and NFKBIA in the TNF signaling pathway, and cause a large amount of secretion of CCL20 in the IL-17 signaling pathway, alleviate inflammatory injury to cells by DPV, inhibit the activity of NF-κB signaling pathway activated by DPV and inhibit the activity of NF-κB signaling pathway activated by DPV, to resist cellular damage and interfere with DPV proliferation in cells.

**Table 4:** Effect of RNA interference NF-κB1 on DEV proliferation

Handle	Group	24 h	36 h	48 h
Infect before you interfere	Experimental group	1.1 × 10^8^*	1.14 × 10^8^	2.67 × 10^8^
	Viral control	6.31 × 10^7^	9.17 × 10^7^	2.04 × 10^8^
	Negative control	2.77 × 10^3^	8.39 × 10^4^	3.43 × 10^4^
	Blank control	4.18 × 10^3^	1.16 × 10^4^	5.58 × 10^3^
Interference before infection	Experimental group	6.44 × 10^6^*	4.52 × 10^7^**	3.53 × 10^7^**
	Viral control	1.14 × 10^7^	8.01 × 10^7^	8.04 × 10^7^
	Negative control	4.81 × 10^3^	9.50 × 10^3^	4.27 × 10^2^
	Blank control	5.04 × 10^3^	1.03 × 10^4^	4.00 × 10^2^
Simultaneous infection and interference	Experimental group	4.28 × 10^7^	3.98 × 10^6^**	6.13 × 10^7^**
	Viral control	4.52 × 10^7^	4.17 × 10^7^	1.43 × 10^8^
	Negative control	4.02 × 10^3^	1.16 × 10^3^	3.85 × 10^3^
	Blank control	1.38 × 10^3^	4.56 × 10^3^	1.96 × 10^3^

In comparison to the virus control group

**Indicates an extremely significant difference (*P* < 0.01)

*Indicates a significant difference (*P* < 0.05), and no marker implies no statistically significant difference

### Effect of NF-κB1 RNAi on DEV proliferation in DEF cells

**Fig. 8:**
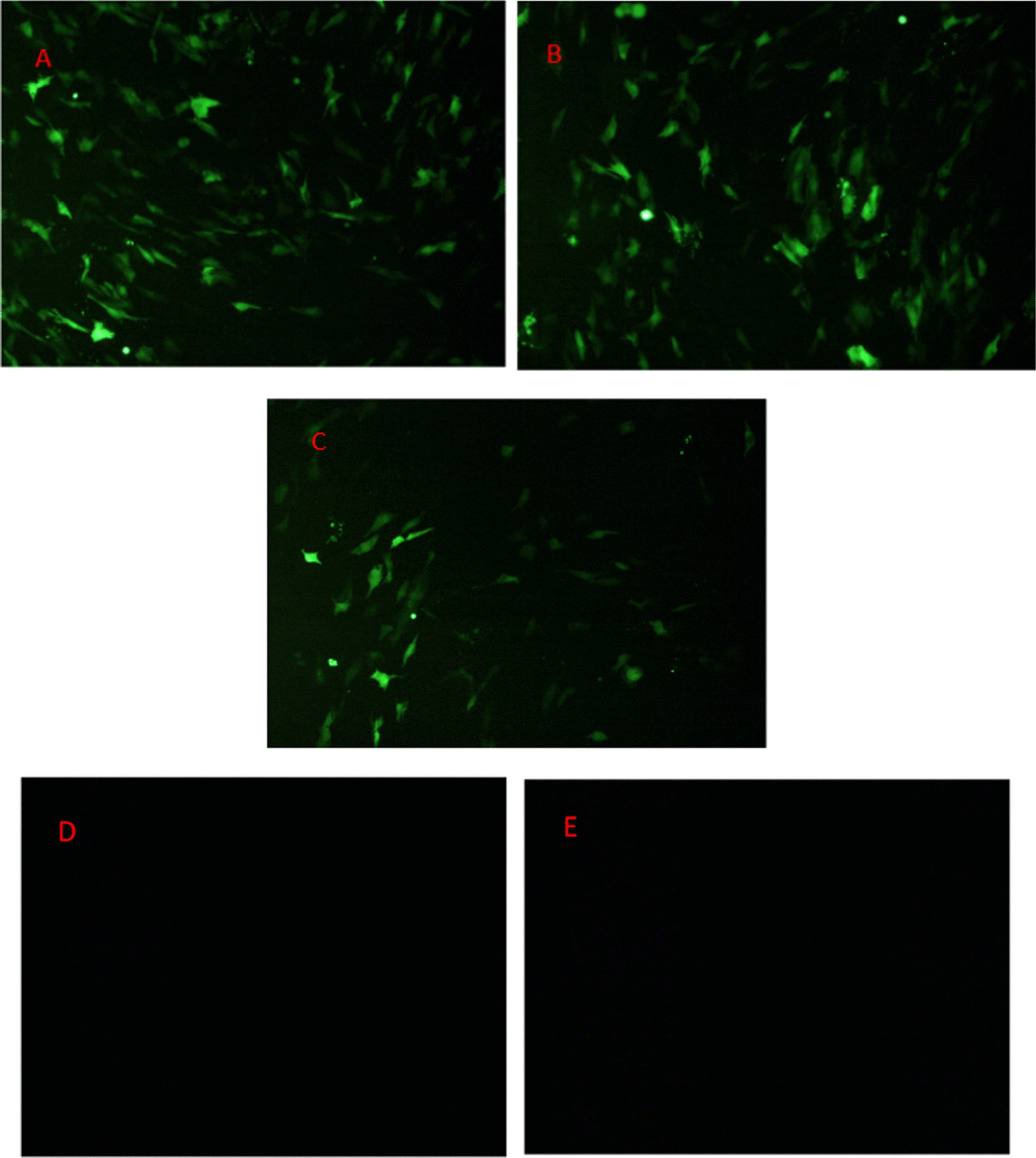
Transfection of pGPU6/GFP/Neo-NF-κB1-2 (100 x)

pGPU6/GFP/Neo-NF-κB-2 and DEV were infected and transfected using three different methods, respectively. Cells were collected at 24, 36, and 48 h, followed by qPCR detection of the DEV-NP gene. The results demonstrated that the group with interference for 24 h followed by treatment with poison achieved an inhibition efficiency of up to 83% at 48 h. Additionally, the group treated with both poison and interference exhibited an inhibition efficiency of up to 77% at 72 h. Notably, the group with interference for 24 h followed by poison treatment showed the highest inhibition efficiency (refer to Table 4, Fig. 8).

### Observations on tissue lesions after DPV infection in ducks

In order to study the effect of DPV-infected ducks on various lymphoid organs, pathological sections were prepared. There were no obvious histopathological changes in various lymphoid organs in group N. In group N, there were no obvious histopathological changes in various lymphoid organs. Splenic organs: Group V showed massive congestion of the splenic sinus at 24 h, nuclear consolidation and nucleolysis at 36 h, and a significant decrease in the number of splenic lymphocytes at 48 h, accompanied by a large number of inflammatory cell infiltration and tissue disintegration (Additional file 5: Fig. S3a, Group V, the arrowheads pointing to the above histopathological lesions); Group M showed an increase in the number of splenic lymphocytes at 24 h, a reduction in the nuclear consolidation and nucleolysis phenomenon at 36 h, and a significant reduction in the hemorrhage or bruising phenomenon of the splenic sinus at 48 h (Additional file 5: Fig. S3a Group M. The arrows point to slight lesions in the above tissues. Thymus: Group V showed eosinophilic staining in both the cortex and medulla at 24 h, with a large number of vacuoles, slight hemorrhage and local inflammatory cell infiltration in the cortex at 36 h, and tissue disintegration and nuclear consolidation in the thymus at 48 h (Additional file 5: Fig. S3b, Group V, the arrowheads pointing to the above histopathology); in Group M, lymphocytosis at 24 h increased in comparison with that of Group V, with a small number of vacuoles at 36 h, and slight inflammatory cell infiltration and lymphocytosis in the cortex and medulla at 48 h. The lymphocytes were increased at 24 h, with a small number of vacuoles at 36 h. In group M, a small number of vacuoles appeared at 48 h compared with group V. At 48 h, the cortex and medulla showed a slight inflammatory cellular infiltration with lymphocytosis, and the histologic lesions did not worsen with the prolongation of the time of infection (Additional file 5: Fig. S3b, group M, the arrowheads pointing to the abovementioned slight histologic lesions). Bursa fascicularis: 24 h markedly reduced basophilic granulocytes in the medulla,36 h nuclear consolidation, nuclear fragmentation, reduced lymphocytes, 48 h tissue disintegration and a small number of hemorrhagic spots (Additional file 5: Fig. S3c Group V, the arrows pointing to the above histopathology); Group M ducks with bursa fascicularis infection 24 h markedly increased basophilic granulocytes in the medulla, 36 h localized irregular fissures and a small amount of hemorrhage, 48 h a slight tissue disintegration (Additional file 5: Fig. S3c Group M, the arrow pointing to the above histopathology). In group M, there was a significant increase in basophils in the medulla of ducks at 24 h after bursa infection. The above results further indicated that honeysuckle could reduce the damage of lymphoid organs caused by DPV infection in ducklings.

### Results of qRT-PCR detection of duck immune organ virus in infected ducks

**Fig. 9:**
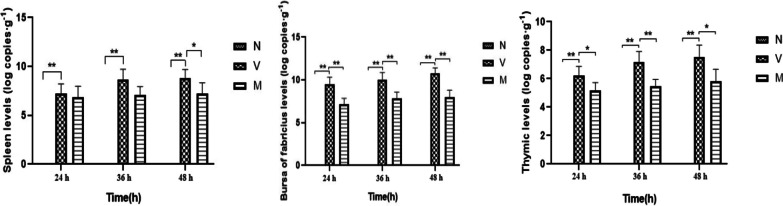
The viral content of each immune organ after DPV infection in ducks

In order to study the actual intervention effect of chlorogenic acid, qPCR was used to detect the viral load in each immune organ of ducks in each group at each time point after DPV infection. As shown in Fig. 9, compared with group N, the DPV load in all lymphoid organs of ducks in group V increased significantly (*P* < 0.05) at all three time points, and compared with group V, the DPV load in all lymphoid organs of ducks in group M decreased. The DPV load of group N was zero in all lymphoid organs. The results showed that chlorogenic acid inhibited the proliferation of DPV in all lymphoid organs of ducklings.

## Discussion

Duck plague virus causes duck viral enteritis in poultry such as ducks, geese and other wild and migratory waterfowl, with high morbidity, mortality and decreased egg production, which seriously affects the healthy development of waterfowl farming [12, 13]. A large number of studies have proved that chlorogenic acid can inhibit the proliferation of adenovirus, hepatitis B virus, herpes simplex virus, dengue virus, etc. in host cells by enhancing the body's immunity, but the specific mechanism is still unclear [14–16]. In this experiment, based on previous studies, chlorogenic acid, an active antiviral component of Honeysuckle, was selected to act on DEF infected with DPV, and based on transcriptomics sequencing, key factors and related metabolic pathways were analyzed to investigate the mechanism of chlorogenic acid to inhibit the proliferation of DPV.

Chemokine ligand 26 (CCL26) interacts with CCR3 to attract eosinophils to sites of inflammation [17] and mediates trans-endothelial migration of eosinophils by binding to and activating G protein-coupled (GPC) chemotaxis of CCR3 on the surface of eosinophils [18]. It was found that significantly elevated levels of the CCL26 gene were found in eosinophilia-related diseases. Sun Xiaochuan [19] et al. collected peripheral blood specimens from patients with primary biliary cholangitis (PBC), isolated plasma and peripheral blood single nucleated cells, and detected plasma CCL26 levels by ELISA, and found that plasma CCL26 levels were significantly elevated in all patients with PBC compared with healthy controls. Tumor necrosis factor α-inducible proteins (TNFAIPs) are a family of TNF-α-induced proteins [20, 21], and its member, TNFAIP2, is a pro-inflammatory cytokine secreted mainly by immune cells [22], which acts as a TNFα-responsive gene regulated by multiple transcription factors and signaling pathways and has the potential to regulate the expression of TNFα. and signaling pathways and has the ability to regulate multiple biological functions including cell proliferation, migration and inflammation [23]. Relevant studies have shown that TNFα will no longer induce TNFAIP2 expression if the NF-κB p65 subunit is deleted, suggesting that TNFAIP2 expression is closely related to the NF-κB pathway [24, 25]. Zhao et al. [26] subjected neuronal cell lines to oxygen–glucose deprivation ( OGD) stress and detected the expression of miR-221, TNF-α, and TNFAIP2 using real-time fluorescence quantitative PCR and immunoblotting, and found that overexpression of TNFAIP2 alleviated miR-221-induced inflammatory response and apoptosis under OGD stress. IFN-1 acts intracellularly by polymerizing the type I IFN receptor (IFNAR1) [27, 28]. It has been shown that neutrophil-mediated regulation of immune responses in lymph nodes is partially regulated by type I IFN signaling. Inactivation of IFNAR1 on neutrophils leads to a significant reduction in neutrophil/T-cell interactions and is accompanied by a decrease in T-cell activity and proliferation [29]. The most obvious way in which IFNAR1 levels influence signaling is by increasing the strength of the signal, as evidenced by the observation that when the concentration of IFNAR1 is increased, IFN-1 binding affinity was significantly increased. suggesting a mechanism by which cells control IFN-1 activity by altering IFNAR1 concentrations [30, 31]. In this experiment, the transcriptomics-based sequencing results after chlorogenic acid and DPV action on DEF revealed that chlorogenic acid caused almost no changes in the expression of DEF cell signaling pathway genes and other related genes except IL-16, and could have an effect on the TNFAIP2, IFNAR1, CCL26, and other related genes IL-16, CHCHD10, CCL26, and other related genes in DEF cells caused by DPV infection, ROR2, and GNG10 expression upregulation and negatively regulate TNFSF15 expression. It suggests that DPV can activate signaling pathways such as NF-κB by up-regulating the content of related pathway factors, which facilitates its own proliferation in cells. Secretion of related inflammatory factors, causing cellular inflammation. Chlorogenic acid can regulate the expression of related genes, inhibit the activation of related pathways, down-regulate the secretion of inflammatory cytokines, alleviate cellular inflammation, and inhibit the proliferation of DPV in DEF cells.

NF-κB is an important transcriptional regulator [32] that has been implicated in many cellular mechanisms, such as immune disorders and inflammatory diseases [33–35]. When organisms are invaded by viruses and other pathogens, NF-κB manipulates certain molecules by targeting host pattern-recognition receptors (PRRs) and viral proteins, receptors recognize many pathogens and their disease-causing components and initiate an innate immune response, and activated TLRs provide an effective defense against pathogens and activate the downstream NF-κB signaling pathway, and the overexpression of TLRs also leads to associated inflammation upregulation of cytokines and genes, exacerbating cellular damage. Drugs block NF-κB subunit gene expression, which in turn inhibits the production of neuroinflammatory mediators or IL -1β-induced inflammatory responses [36]. Liao Shanghui et al. [37] found that chlorogenic acid inhibited the expression of NF-κB P105 protein in the lung tissues of influenza virus-infected mice, suggesting that chlorogenic acid may exert its anti-influenza virus effect by inhibiting the NF-κB signaling pathway. In this experiment, transcriptome-based sequencing of DPV-infected DEF after chlorogenic acid action revealed that differentially expressed genes were mainly present 36 h after chlorogenic acid action, and a large number of related pathway genes were up-regulated or down-regulated at this time. The invasion of DEF by DPV led to the activation of TLR4, which triggered the downstream NF-κB signaling pathway, regulating the production of cytokines and the expression of many inflammatory genes. expression. Transcriptome sequencing results showed that TLR4 expression was suppressed in DPV-infected DEF after exposure to chlorogenic acid. Thus, chlorogenic acid impairs inflammation-mediated signaling to downstream signaling pathways. NFKBIA, an upstream regulator of NF-κB activity, inhibits NF-κB activation by binding to NF-κB in the cytoplasm and inhibiting its translocation to the nucleus [38]. Normally, the NF-κB complex exists in the cytoplasm in an inactive form through the inhibitor IκB protein, which is phosphorylated and degraded by the action of the IκB kinase (IKK) complex (e.g., IKKα, IKKβ) upon cellular stimulation; with the degradation of the IκB protein, NF-κB is released for translocation to the nucleus and activates the transcription of a large number of genes [39]. In this study, we found that the activities of TOLL-like receptor pathway and NF-κB signaling pathway were inhibited after chlorogenic acid acted on DPV-infected DEF, which may be attributed to the fact that chlorogenic acid inhibited DPV-induced phosphorylation of NF-κB P65 protein with its inhibitor IkBα by down-regulating DPV-induced cyclo-oxygenase 2 (COX-2) and TLR4 expression and up-regulated NFKBIA expression, thereby inhibiting DPV proliferation in DEF with NF-κB signaling pathway-mediated inflammation damaging DEF. The same regulatory mechanism was found in the TNF signaling pathway, suggesting that the effect of chlorogenic acid on DPV is closely related to NF-κB. This is consistent with the findings of many scholars.

The spleen, thymus and bursa, as important components of the avian lymphatic system, are important lymphoid organs for generating an immune response after pathogen invasion into the organism [40, 41]. The lymphatic system mainly consists of T cells and B cells, T cells differentiate and mature in the thymus to participate in the cellular immune response, and B cells in the bursa of Fasciola to participate in the humoral immune response, in the spleen, T cells account for 35–50%, and B cells account for 50–60%, and these two types of cells play a key role in the resistance to the invasion of the virus [42]. Studies have shown that DEV-infected ducklings show different degrees of damage to tissues and organs, manifested by irreversible pathological changes such as rupture of the endothelium of the wall tubes, reduction of lymphocytes in the central organs, and obvious cellular degeneration in various organs [43]. In this study, we focused on the histopathological changes and viral load of lymphoid organs after experimental infection of ducks with DPV-GZ strain. The most obvious and consistent gross lesions observed in these lymphoid organs were hemorrhage, congestion, tissue disintegration, inflammatory cell infiltration, nuclear consolidation and nuclear fragmentation, etc. The above histopathological lesions were reduced to different degrees after intervention with chlorogenic acid, and the results of qPCR assay showed that chlorogenic acid could inhibit the proliferation of DPV in the lymphoid organs of ducklings. The results of qPCR also showed that chlorogenic acid could inhibit the proliferation of DPV in the lymphoid organs of ducklings, which indicated that chlorogenic acid could help ducklings to resist the attack of DPV on the lymphoid organs and alleviate the damage caused by DPV on the lymphoid organs of ducklings.

In this experiment, transcriptome sequencing revealed that DPV infection of DEF mainly caused down-regulation of differential genes, and mainly caused up-regulation of differential genes under the intervention of chlorogenic acid, suggesting that DPV may regulate and inhibit the expression of relevant genes when infecting cells in order to facilitate its own proliferation within the host cells, whereas chlorogenic acid inhibits this effect by up-regulating the expression of relevant genes, thus affecting the proliferation of DPV in the organism.

## Conclusion

The NF-κB signaling pathway plays an important role in DEV infection with DEF. KEGG enrichment analysis showed that differentially expressed genes are highly enriched in the NF-κB signaling pathway. When NF-κB1 gene is silenced, the copy number of DEV decreases, indicating that chlorogenic acid can inhibit viral replication by regulating the NF-κB signaling pathway. Thus exert the antiviral effect. In addition, chlorogenic acid in vivo can also reduce the load of DEV in ducks and alleviate the damage of duck lymphatic organs. This study provides a theoretical basis for screening anti-Dev targeted drugs.

### Supplementary Information

**Additional file 1. Fig. S1**. Volcano map comparing differences of each group.

**Additional file 2. Fig. S2.** qRT-PCR verification results of differential genes.

**Additional file 3. Fig. S3**. Pathological observations on the immune organs of ducks infected with duck plague virus.

**Additional file 4. Table S1**. Primer sequences used for qPCR.

**Additional file 5. Table S2.** Filtering statistics for transcriptome sequencing data.

## References

[1] Li Y, Wang M, Cheng A, Jia R, Yang Q, Chen S, Zhu D, Liu M, Zhao X, Zhang S (2020). Duck enteritis virus VP16 antagonizes ifn-beta-mediated antiviral innate immunity. J Immunol Res.

[2] Yang L, Shen B, Wang M, Cheng A, Yang Q, Wu Y, Huang J, Tian B, Jia R, Liu M (2021). The intracellular domain of duck plague virus glycoprotein E affects UL11 protein incorporation into viral particles. Vet Microbiol.

[3] Huang YL: Effects of intracellular and extracellular domains of duck plague virus capsule glycoprotein gE on virus virulence and immune efficacy. Sichuan Agricultural University, 2020.

[4] Proctor SJ (1976). Pathogenesis of duck plague in the bursa of Fabricius, thymus, and spleen. Am J Vet Res.

[5] Li C, Wang M, Cheng A, Tian B, Huang J, Wu Y, Yang Q, Gao Q, Sun D, Zhang S, et al. Deleting UL495 in duck plague virus causes attachment, entry and spread defects. Vet Microbiol 2023;280:109707. 10.1016/j.vetmic.2023.109707 36863173

[6] Cheng C, Zhang W (2021). Shi L Yun: Research progress of antiviral components of traditional Chinese medicine and their molecular targets. Chin J Tradit Chin Med.

[7] Dhama K, Kumar N, Saminathan M, Tiwari R, Karthik K, Kumar MA, Palanivelu M, Shabbir MZ, Malik YS, Singh RK (2017). Duck virus enteritis (duck plague): a comprehensive update. Vet Q.

[8] Wang GF, Shi LP, Ren YD, Liu QF, Liu HF, Zhang RJ, Li Z, Zhu FH, He PL, Tang W (2009). Anti-hepatitis B virus activity of chlorogenic acid, quinic acid and caffeic acid in vivo and in vitro. Antiviral Res.

[9] Li Y, Yang D, Jia Y, He L, Li J, Yu C, Liao C, Yu Z, Zhang C (2021). Research Note: Anti-inflammatory effects and antiviral activities of baicalein and chlorogenic acid against infectious bursal disease virus in embryonic eggs. Poult Sci.

[10] Li X, Liu Y, Hou X, Peng H, Zhang L, Jiang Q, Shi M, Ji Y, Wang Y, Shi W (2013). Chlorogenic acid inhibits the replication and viability of enterovirus 71 in vitro. PLoS ONE.

[11] Zhang QD, Bi WW, Zhang Y, Li T, Zhang P, Wen GL, Yang Y, Cheng ZT, Wen M (2022). Effects of honeysuckle on immune organ function in ducks infected with duck plague virus. Chin J Prev Vet Med.

[12] Wu Y, Zhang S, Li Y, Pan C, Wang M, Chen S, Jia R, Yang Q, Zhu D, Liu M (2023). Establishment and application of a PCR assay for the identification of virulent and attenuated duck plague virus DNA in cotton swabs. Poult Sci.

[13] Yang J, Song SL, Castro-Perez J, Plumb RS, Xu GW (2005). Metabonomics and its applications. Sheng Wu Gong Cheng Xue Bao.

[14] Ge L, Xiao L, Wan H, Li J, Lv K, Peng S, Zhou B, Li T, Zeng X (2019). Chemical constituents from Lonicera japonica flower buds and their anti-hepatoma and anti-HBV activities. Bioorg Chem.

[15] Jose-Abrego A, Rivera-Iniguez I, Torres-Reyes LA, Roman S (2023). Anti-hepatitis B virus activity of food nutrients and potential mechanisms of action. Ann Hepatol.

[16] Fredsgaard M, Kaniki S, Antonopoulou I, Chaturvedi T, Thomsen MH. Phenolic compounds in salicornia spp. and their potential therapeutic effects on H1N1, HBV, HCV, and HIV: a review. Molecules 2023;28. 10.3390/molecules28145312 PMC10384198 37513186

[17] Lan Q, Lai W, Zeng Y, Liu L, Li S, Jin S, Zhang Y, Luo X, Xu H, Lin X, Chu Z (2018). CCL26 participates in the PRL-3-induced promotion of colorectal cancer invasion by stimulating tumor-associated macrophage infiltration. Mol Cancer Ther.

[18] Pum A, Ennemoser M, Gerlza T, Kungl AJ. The role of Heparan sulfate in CCL26-induced eosinophil chemotaxis. Int J Mol Sci 2022;23. 10.3390/ijms23126519 PMC9224159 35742962

[19] Sun XC. The role of CX3CL1/CCL26-CX3CR1 pathway in the pathogenesis of primary biliary cholangitis. Peking Union Medical College 2019.

[20] Guo F, Yuan Y (2020). Tumor necrosis factor alpha-induced proteins in malignant tumors: progress and prospects. Onco Targets Ther.

[21] Lan G, Yu X, Sun X, Li W, Zhao Y, Lan J, Wu X, Gao R (2021). Comprehensive analysis of the expression and prognosis for TNFAIPs in head and neck cancer. Sci Rep.

[22] Lin MS, Zhong HY, Yim RL, Chen QY, Du HL, He HQ, Lin K, Zhao P, Gao R, Gao F, Zhang MY (2022). Pan-cancer analysis of oncogenic TNFAIP2 identifying its prognostic value and immunological function in acute myeloid leukemia. BMC Cancer.

[23] Xie Y, Wang B (2017). Downregulation of TNFAIP2 suppresses proliferation and metastasis in esophageal squamous cell carcinoma through activation of the Wnt/beta-catenin signaling pathway. Oncol Rep.

[24] Jin GY. Mechanism of TNFAIP2 regulating macrophage function and promoting atherosclerosis. Shandong University 2019.

[25] Jia L, Shi Y, Wen Y, Li W, Feng J, Chen C (2018). The roles of TNFAIP2 in cancers and infectious diseases. J Cell Mol Med.

[26] Zhao D, Deng SC, Ma Y, Hao YH, Jia ZH (2018). miR-221 alleviates the inflammatory response and cell apoptosis of neuronal cell through targeting TNFAIP2 in spinal cord ischemia-reperfusion. NeuroReport.

[27] Phillips MB, Dina ZM, Howells MA, Weinkopff T, Boehme KW. Lymphatic type 1 interferon responses are critical for control of systemic reovirus dissemination. J Virol 2021;95. 10.1128/JVI.02167-20 PMC7851543 33208448

[28] Schreiber G (2017). The molecular basis for differential type I interferon signaling. J Biol Chem.

[29] Hussain T, Domnich M, Bordbari S, Pylaeva E, Siakaeva E, Spyra I, Ozel I, Droege F, Squire A, Lienenklaus S (2022). IFNAR1 deficiency impairs immunostimulatory properties of neutrophils in tumor-draining lymph nodes. Front Immunol.

[30] Antunes KH, Fachi JL, de Paula R, Da SE, Pral LP, Dos SA, Dias G, Vargas JE, Puga R, Mayer FQ (2019). Microbiota-derived acetate protects against respiratory syncytial virus infection through a GPR43-type 1 interferon response. Nat Commun.

[31] Keller N, Woytschak J, Heeb L, Marques ME, Mairpady SS, Snall J, Hyldegaard O, Boyman O, Norrby-Teglund A, Zinkernagel AS (2019). Group A streptococcal dnase sda1 impairs plasmacytoid dendritic cells' type 1 interferon response. J Invest Dermatol.

[32] Mitchell JP, Carmody RJ (2018). NF-kappaB and the transcriptional control of inflammation. Int Rev Cell Mol Biol.

[33] Barnabei L, Laplantine E, Mbongo W, Rieux-Laucat F, Weil R (2021). NF-kappaB: at the borders of autoimmunity and inflammation. Front Immunol.

[34] Kaltschmidt C, Greiner J, Kaltschmidt B. The Transcription factor NF-kappaB in stem cells and development. Cells 2021;10. 10.3390/cells10082042 PMC8391683 34440811

[35] Li Z, Wang S, Han J, Shi C, Yang G, Cui Y, Xi L, Yin S, Zhang H (2023). Deletion of Brucella transcriptional regulator GntR10 regulated the expression of quorum sensing system and type IV secretion system effectors, which affected the activation of NF-kappaB. J Proteomics.

[36] Song FJ, Zeng KW, Chen JF, Li Y, Song XM, Tu PF, Wang XM (2019). Extract of Fructus Schisandrae chinensis Inhibits Neuroinflammation Mediator Production from Microglia via NF-kappa B and MAPK Pathways. Chin J Integr Med.

[37] Liao SH. Study on the anti-influenza effect of chlorogenic acid, the main component of Yinqiao Powder, and its related signaling pathway molecular mechanism. Guangzhou University of Traditional Chinese Medicine 2019.

[38] Yang W, Li J, Zhang M, Yu H, Zhuang Y, Zhao L, Ren L, Gong J, Bi H, Zeng L (2022). Elevated expression of the rhythm gene NFIL3 promotes the progression of TNBC by activating NF-kappaB signaling through suppression of NFKBIA transcription. J Exp Clin Cancer Res.

[39] Fan W, Liu X, Zhang J, Qin L, Du J, Li X, Qian S, Chen H, Qian P (2022). TRIM67 suppresses TNFalpha-triggered NF-kB activation by competitively binding beta-TrCP to IkBa. Front Immunol.

[40] Cooper MD, Raymond DA, Peterson RD, South MA, Good RA (1966). The functions of the thymus system and the bursa system in the chicken. J Exp Med.

[41] Islam R, Sultana N, Haque Z, Rafiqul IM (2023). Effect of dietary dexamethasone on the morphologic and morphometric adaptations in the lymphoid organs and mortality rate in broilers. Vet Med Sci.

[42] Cui H, Jing F, Xi P (2003). Pathology of the thymus, spleen and bursa of Fabricius in zinc-deficient ducklings. Avian Pathol.

[43] Shawky S (2000). Target cells for duck enteritis virus in lymphoid organs. Avian Pathol.

