# Supplementary material for: RNA-seq analysis of chlorogenic acid intervention in duck embryo fibroblasts infected with duck plague virus
Source: Virol J. 2024 Mar 7;21:60. doi: 10.1186/s12985-024-02312-2 (PMC10921813; doi:10.1186/s12985-024-02312-2)
Supplement: Supplementary file 1 — Additional file 1. Fig. S1. Volcano map comparing differences of each group. [file 12985_2024_2312_MOESM1_ESM.docx]

Table.S1. Primer sequences used for qPCR

| Genes | Primer sequence (5'---3′) |
| --- | --- |
| *IL-16* | F:AGCACAGCTAGTGCACACAT  R:GCAGCTCCACCAGGAAAGAT |
| *TNFAIP2* | F:CAGCTGGAAAACCCTGCAAC  R:GCCTCTGGCCTTTCACTCAT |
| *IFN-AR1* | F:GGAAGGAGTTGCCTGGTTGT  R:TACGCGCACAAGATGCCTAT |
| *CCL26* | F:TGCTGGCCTCTTTTCTCAGG  R:AGGTTTAGCACAGACCTGCC |
| *TNFSF15* | F:GCTTAGGAGACAAAGCGGGT  R:TCTGACAGCTGCTGAGTGTG |
| *CHCHD10* | F:GCTGAAGCAGTGCAAGTACA  R:GGCATCAGGGCTCTACACC |
| *ROR2* | F:GAGGGACAGTCAGCGTTACC  R:GCATGTCCTCCACCTAGCTC |
| *GNG10* | F:GAGAGCCTGCCTTCAAGCAT  R:AGGTAGTGAGGTACGGGTGC |
| *DPV -NP* | F:CTGGAAGATGCAGTAACGTCTG  R:CTGGGTTGTCTGTATTCGGAGT |
| *β-Actin* | F:CTACAGCTTCACCACCACAGCC  R:GCTGTGGCCATCTCCTGCTCAA |
