# Supplementary material for: RNA-seq analysis of chlorogenic acid intervention in duck embryo fibroblasts infected with duck plague virus
Source: Virol J. 2024 Mar 7;21:60. doi: 10.1186/s12985-024-02312-2 (PMC10921813; doi:10.1186/s12985-024-02312-2)
Supplement: Supplementary file 2 — Additional file 2. Fig. S2. qRT-PCR verification results of differential genes. [file 12985_2024_2312_MOESM2_ESM.docx]

**Table S1 Filtering statistics for transcriptome sequencing data**

| Sample | Total Raw Reads (M) | Total Clean Reads (M) | Total Clean Bases (Gb) | Clean Reads Q20 (%) | Clean Reads Q30 (%) | Clean Reads Ratio (%) |
| --- | --- | --- | --- | --- | --- | --- |
| A_24_1 | 47.33 | 45.08 | 6.76 | 97.8 | 93.77 | 95.26 |
| A_24_2 | 47.33 | 44.72 | 6.71 | 97.87 | 93.92 | 94.49 |
| A_24_3 | 47.33 | 44.86 | 6.73 | 97.89 | 94.05 | 94.79 |
| A_36_1 | 47.33 | 45.08 | 6.76 | 97.38 | 93.01 | 95.25 |
| A_36_2 | 47.33 | 44.79 | 6.72 | 97.4 | 93.11 | 94.65 |
| A_36_3 | 47.33 | 44.1 | 6.61 | 97.61 | 93.67 | 93.18 |
| A_48_1 | 47.33 | 44.88 | 6.73 | 97.53 | 93.42 | 94.82 |
| A_48_2 | 47.33 | 45.5 | 6.83 | 97.41 | 93.06 | 96.14 |
| A_48_3 | 47.33 | 44.66 | 6.7 | 97.43 | 93.23 | 94.37 |
| C_24_1 | 47.33 | 45.15 | 6.77 | 97.76 | 93.65 | 95.39 |
| C_24_2 | 47.33 | 45.1 | 6.77 | 97.87 | 93.95 | 95.3 |
| C_24_3 | 47.33 | 44.66 | 6.7 | 97.68 | 93.81 | 94.36 |
| C_36_1 | 47.33 | 45.33 | 6.8 | 97.5 | 93.28 | 95.78 |
| C_36_2 | 47.33 | 45.29 | 6.79 | 97.61 | 93.55 | 95.69 |
| C_36_3 | 47.33 | 44.41 | 6.66 | 97.57 | 93.55 | 93.83 |
| C_48_1 | 50.83 | 45.39 | 6.81 | 97.58 | 93.62 | 89.29 |
| C_48_2 | 47.33 | 44.69 | 6.7 | 97.35 | 93.06 | 94.42 |
| C_48_3 | 47.33 | 44.85 | 6.73 | 97.4 | 93.12 | 94.76 |
| D_24_1 | 47.33 | 44.68 | 6.7 | 97.25 | 92.74 | 94.4 |
| D_24_2 | 47.33 | 44.63 | 6.7 | 97.95 | 94.18 | 94.31 |
| D_24_3 | 47.33 | 44.63 | 6.69 | 97.91 | 94.07 | 94.31 |
| D_36_1 | 47.33 | 44.27 | 6.64 | 97.43 | 93.19 | 93.53 |
| D_36_2 | 47.33 | 45.24 | 6.79 | 97.31 | 92.81 | 95.59 |
| D_36_3 | 47.33 | 44.28 | 6.64 | 97.4 | 93.19 | 93.56 |
| D_48_1 | 47.33 | 45.03 | 6.76 | 97.25 | 92.78 | 95.16 |
| D_48_2 | 47.33 | 44.93 | 6.74 | 97.34 | 93 | 94.95 |
| D_48_3 | 47.33 | 45.05 | 6.76 | 97.39 | 93.16 | 95.2 |
| F_24_1 | 47.33 | 44.8 | 6.72 | 97.59 | 93.64 | 94.65 |
| F_24_2 | 47.33 | 44.93 | 6.74 | 97.43 | 93.14 | 94.93 |
| F_24_3 | 47.33 | 44.68 | 6.7 | 97.36 | 93.02 | 94.4 |
| F_36_1 | 47.33 | 44.86 | 6.73 | 97.79 | 93.82 | 94.78 |
| F_36_2 | 47.33 | 44.75 | 6.71 | 98 | 94.38 | 94.56 |
| F_36_3 | 47.33 | 44.44 | 6.67 | 97.55 | 93.46 | 93.9 |
| F_48_1 | 47.33 | 44.85 | 6.73 | 97.48 | 93.34 | 94.77 |
| F_48_2 | 47.33 | 44.55 | 6.68 | 97.59 | 93.62 | 94.13 |
| F_48_3 | 47.33 | 45.18 | 6.78 | 97.68 | 93.48 | 95.46 |

Total Raw Reads (M): number of reads before filtration; Total Clean Reads (M): number of reads after filtration; Total Clean Bases (Gb): number of bases after filtration; Clean Reads Q20 (%): percentage of bases with a quality value of more than 20 in the filtered reads; Clean Reads Q30 (%): percentage of bases with a quality value of more than 30 in the filtered reads; Clean Reads Ratio (%): percentage of bases with a quality value of more than 30 in the filtered reads; Clean Reads Ratio (%): percentage of bases in the filtered reads Clean Reads Q30(%): the percentage of total bases in filtered reads with quality value greater than 30; Clean Reads Ratio(%): the proportion of filtered reads.
