# Supplementary material for: RNA-seq analysis of chlorogenic acid intervention in duck embryo fibroblasts infected with duck plague virus
Source: Virol J. 2024 Mar 7;21:60. doi: 10.1186/s12985-024-02312-2 (PMC10921813; doi:10.1186/s12985-024-02312-2)
Supplement: Supplementary file 3 — Additional file 3. Fig. S3. Pathological observations on the immune organs of ducks infected with duck plague virus. [file 12985_2024_2312_MOESM3_ESM.docx]

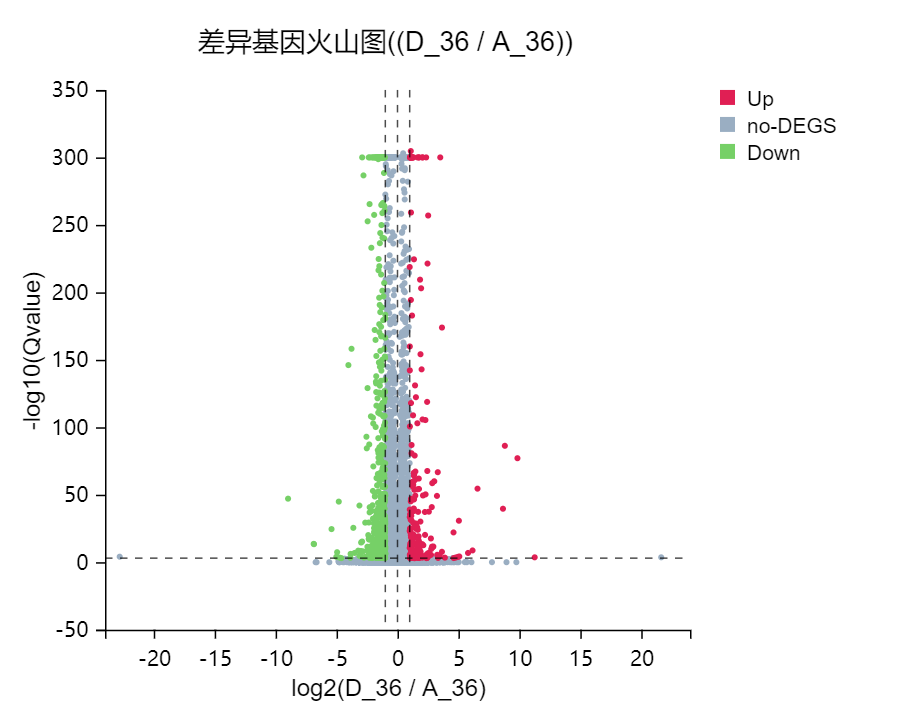


A36 VS D36


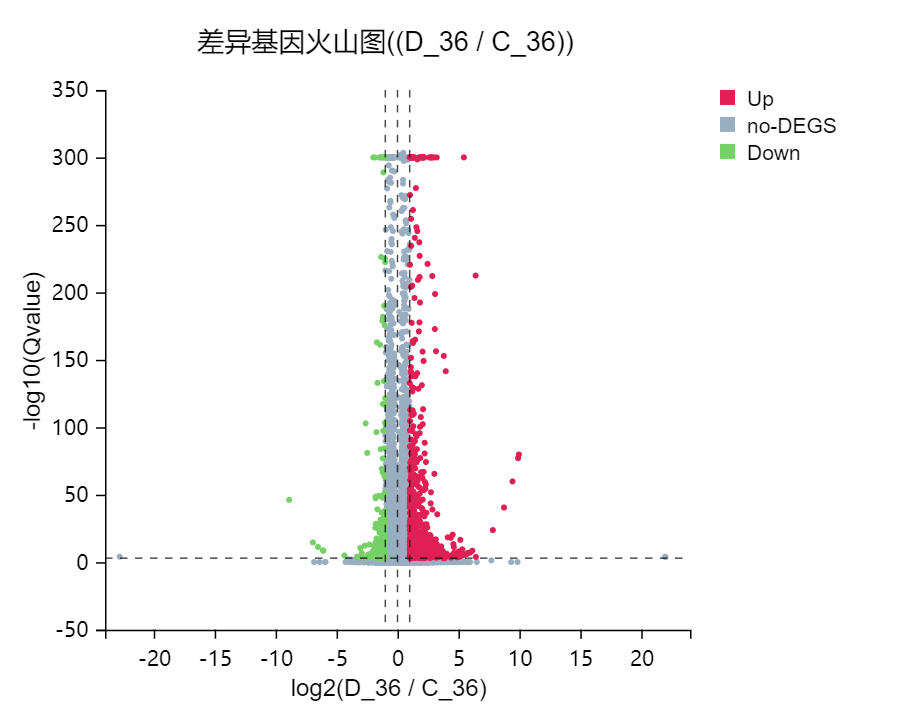


C36 VS D36


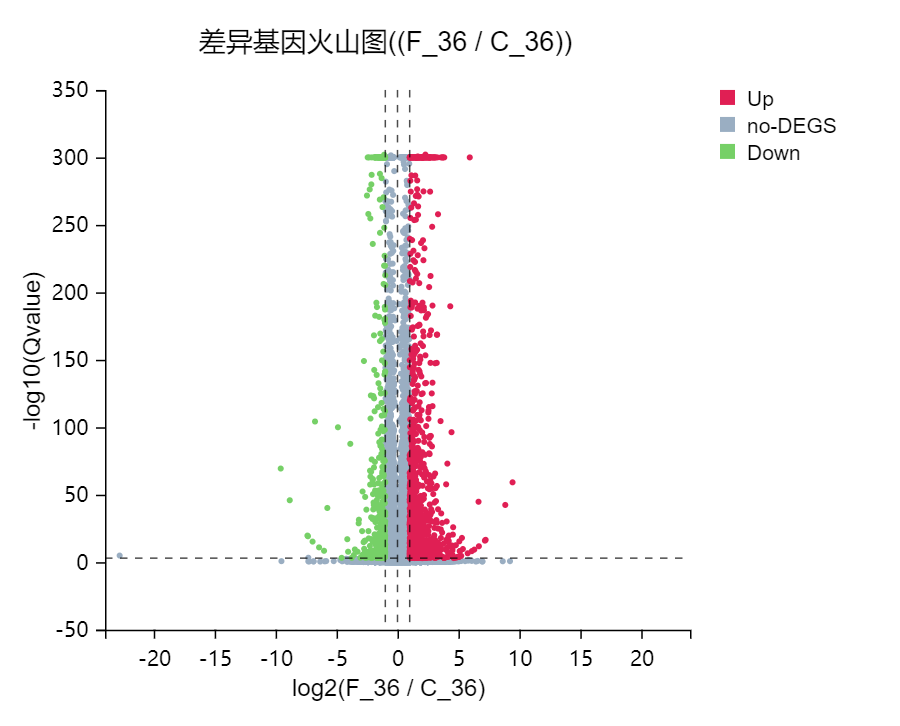


C36 VS F36


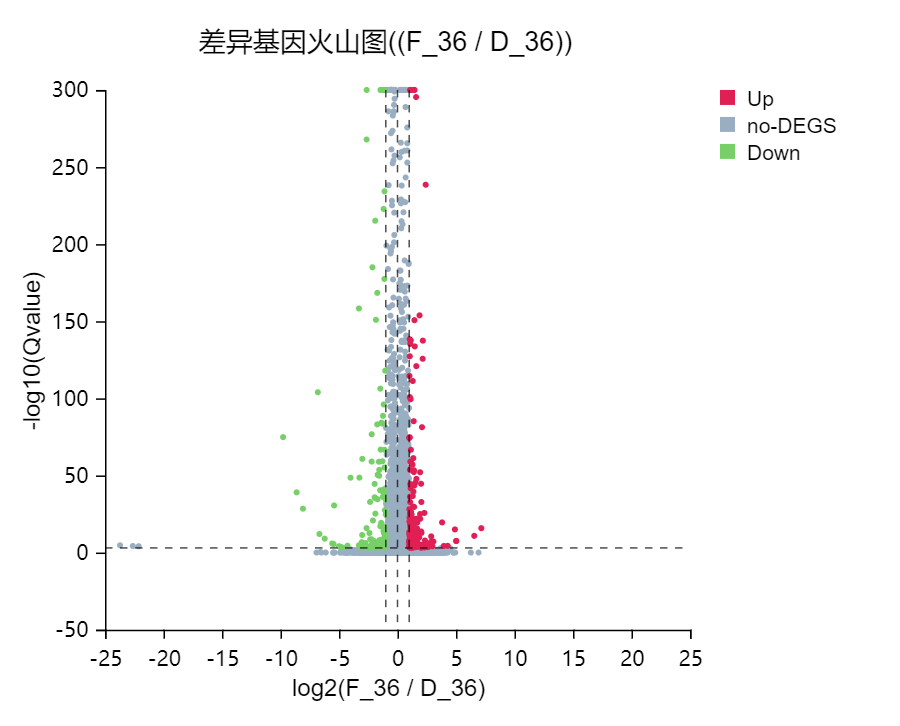


D36 VS F36


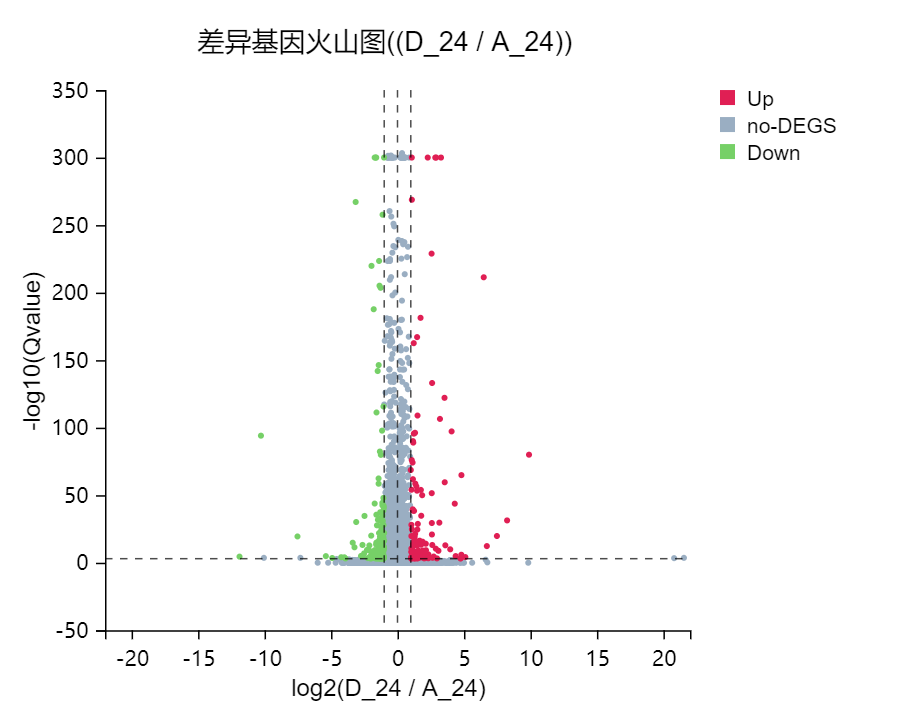


A24 VS D24


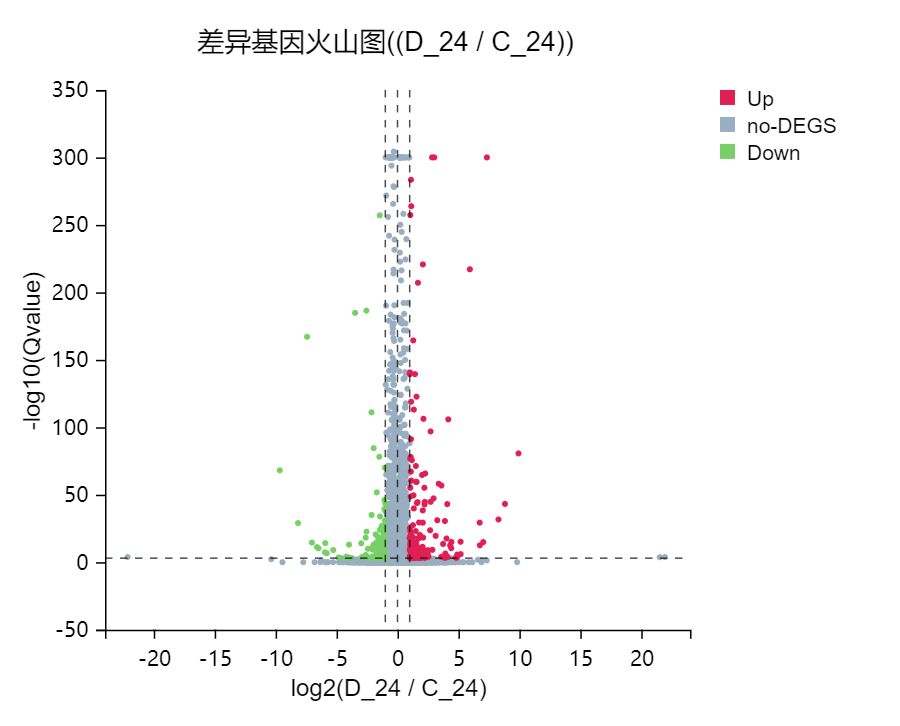


C24 VS D24


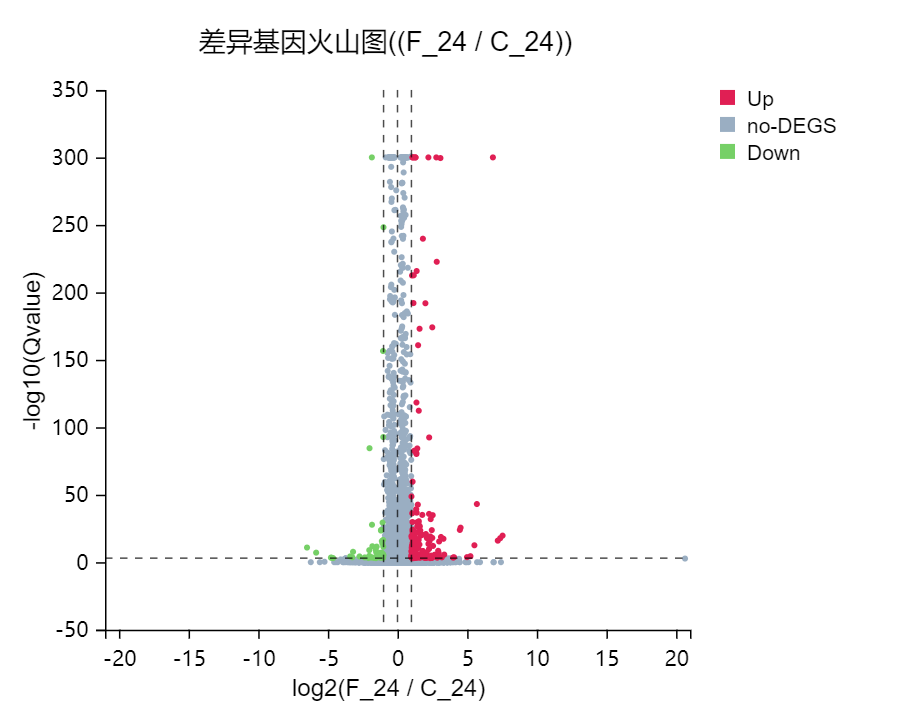


C24 VS F24

D24 VS F24


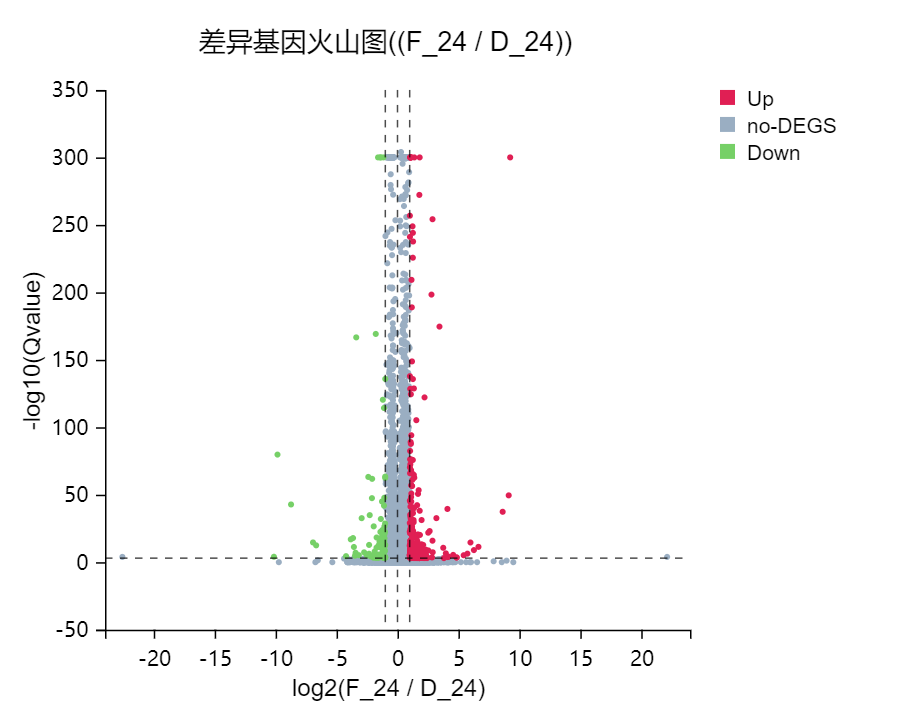

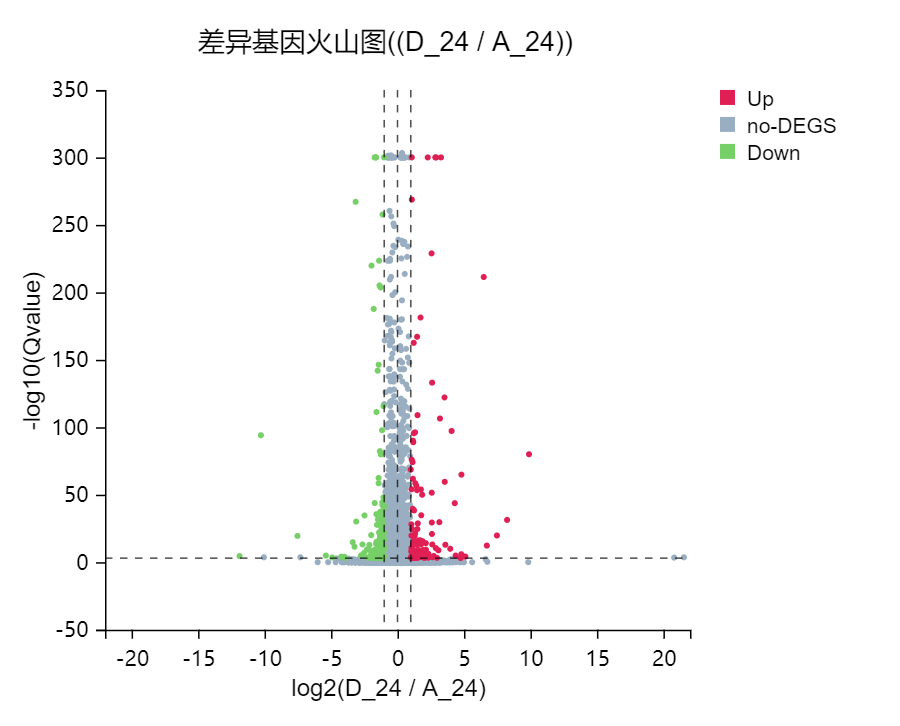


A48 VS D48

C48 VS D48


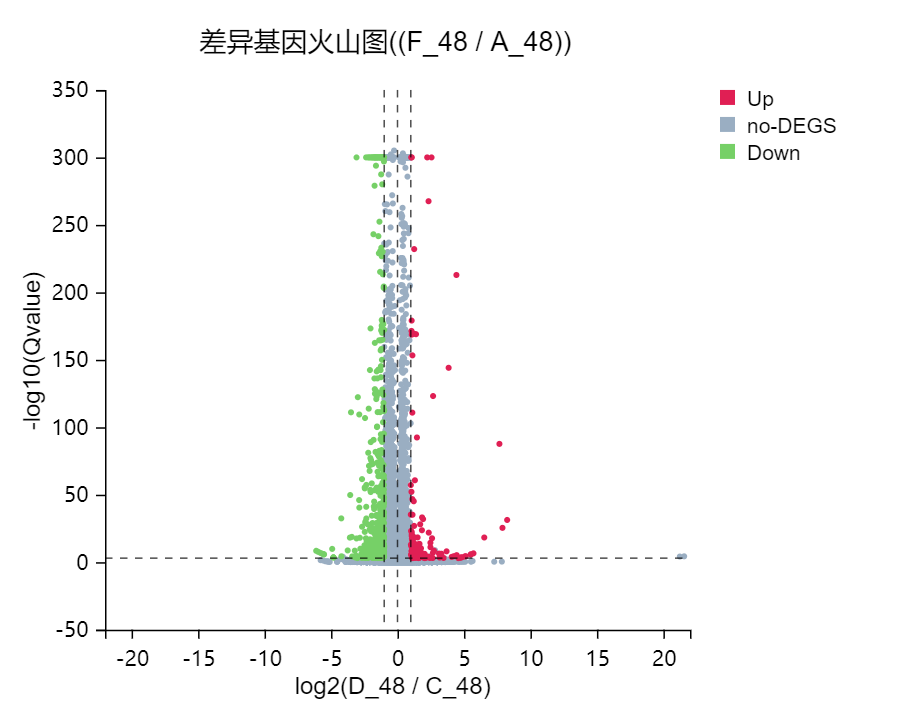


C48 VS F48


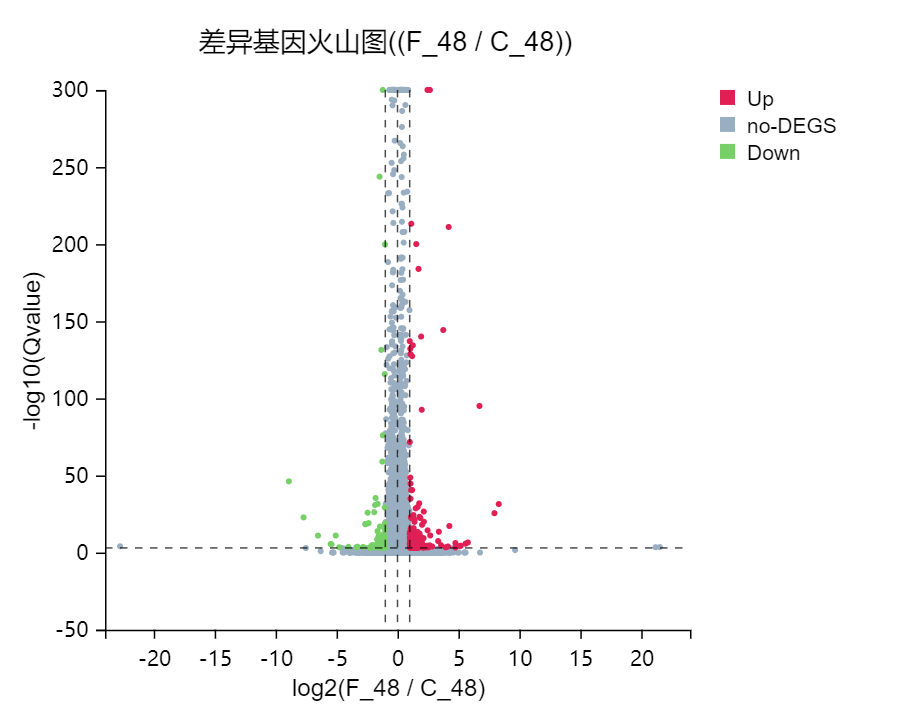


D48 VS F48


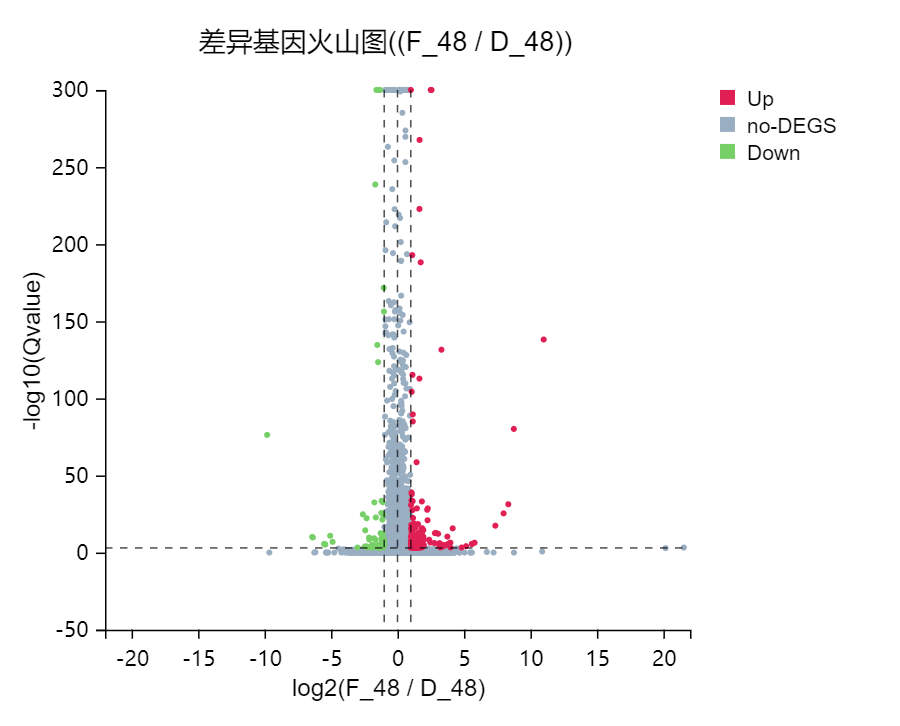


**Fig. S1. Volcano map comparing differences of each group**
