# Supplementary figures and images for: RNA-seq analysis of chlorogenic acid intervention in duck embryo fibroblasts infected with duck plague virus
Source: Virol J. 2024 Mar 7;21:60. doi: 10.1186/s12985-024-02312-2 (PMC10921813; doi:10.1186/s12985-024-02312-2)

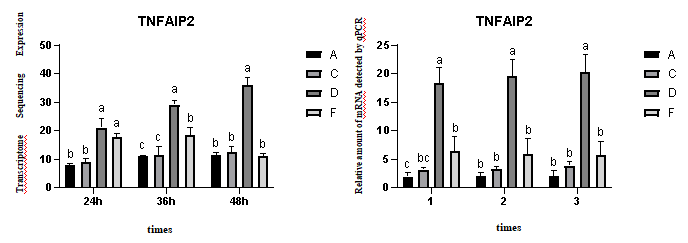


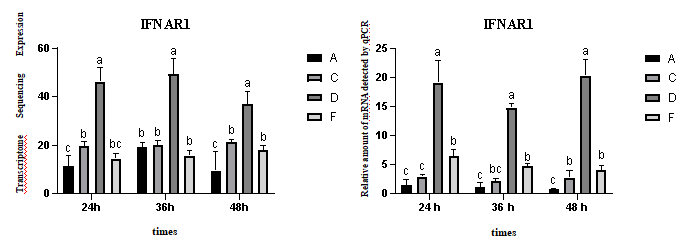


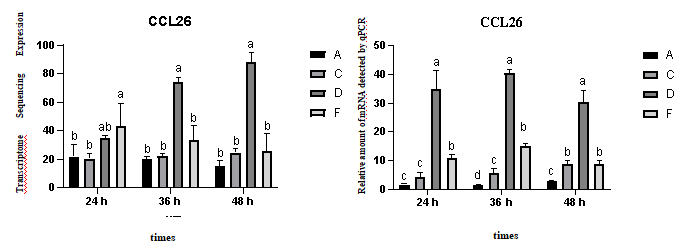


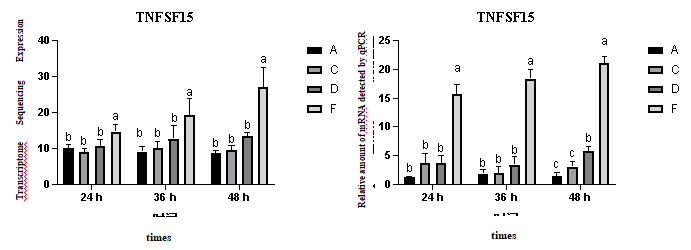


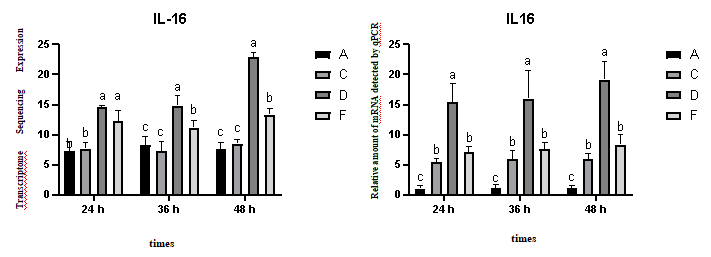


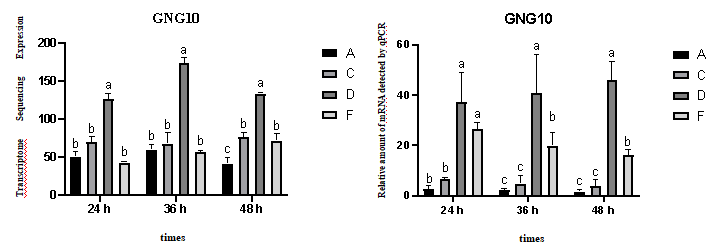


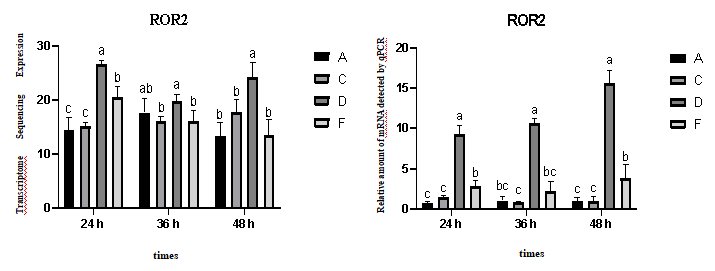

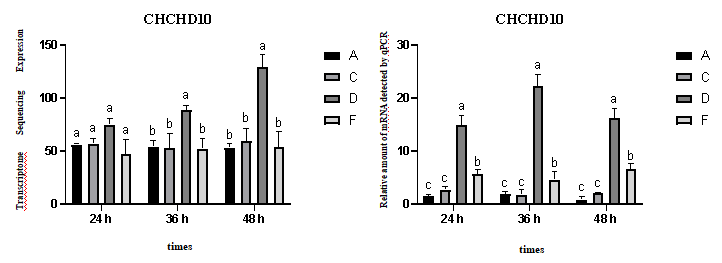


**Fig. S2. qRT-PCR verification results of differential genes**

Supplement: Supplementary file 4 — Additional file 4. Table S1. Primer sequences used for qPCR. [file 12985_2024_2312_MOESM4_ESM.docx]
