# Supplementary material for: RNA-seq analysis of chlorogenic acid intervention in duck embryo fibroblasts infected with duck plague virus
Source: Virol J. 2024 Mar 7;21:60. doi: 10.1186/s12985-024-02312-2 (PMC10921813; doi:10.1186/s12985-024-02312-2)
Supplement: Supplementary file 5 — Additional file 5. Table S2. Filtering statistics for transcriptome sequencing data. [file 12985_2024_2312_MOESM5_ESM.docx]

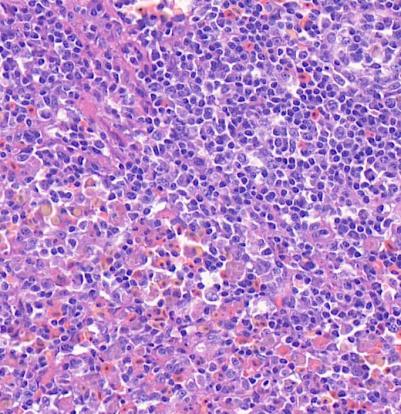

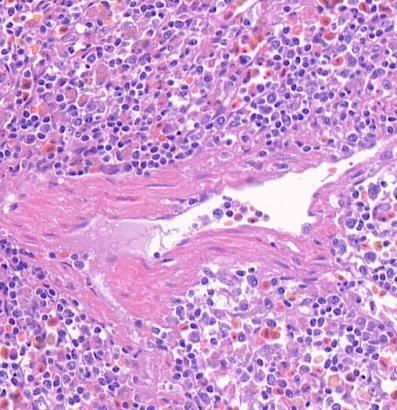

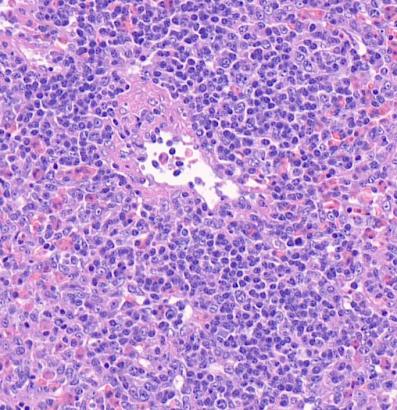


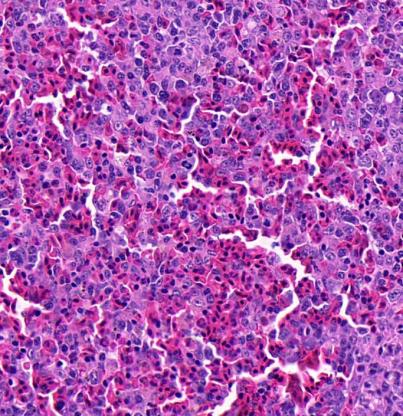

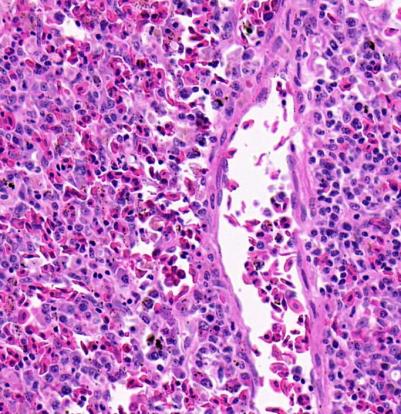

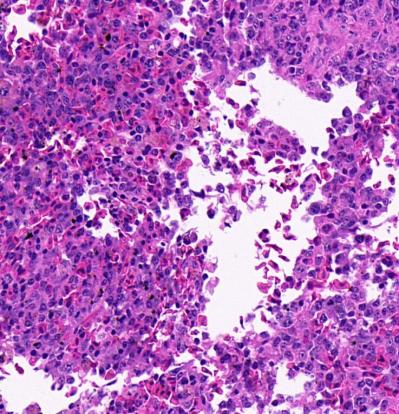


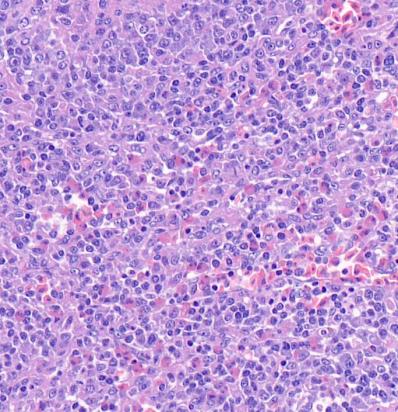

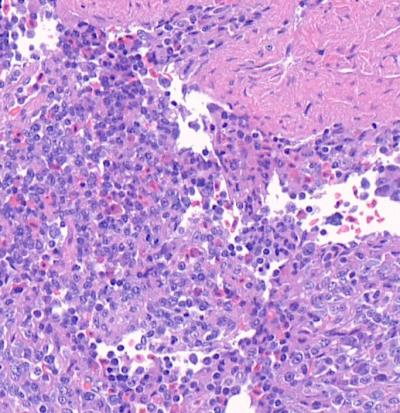

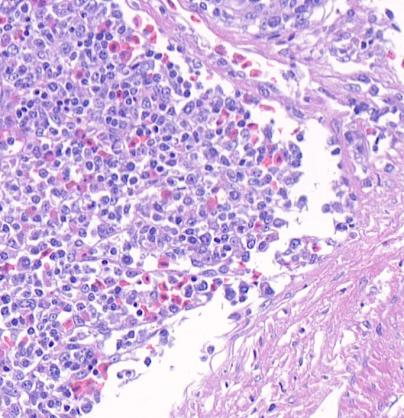


**Group N**

**Group V**

**Group M**

Fig.S3a. Pathological observation of spleen tissue of ducks infected with duck distemper virus（40×）.(N). Normal control group; (V).virus infected group;( M). Drug intervention group

**24 h**

**36 h**

**48 h**

a

**48 h**

**36 h**

**24 h**

**Group N**

**Group V**

**Group M**

Fig.S3b. Gross pathological observation of thymus of ducks infected with duck distemper virus（40×）. (N).Normal control group; (V).virus infected group; (M). Drug intervention group


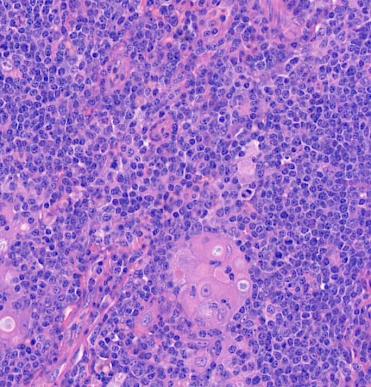

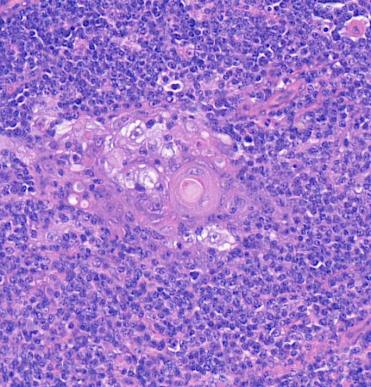

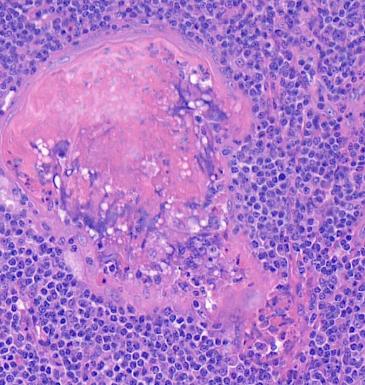

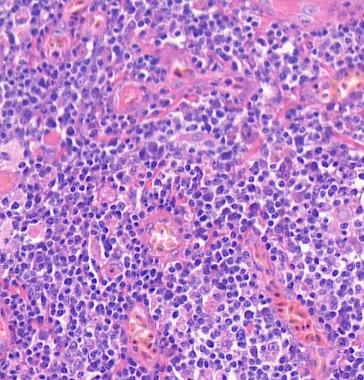

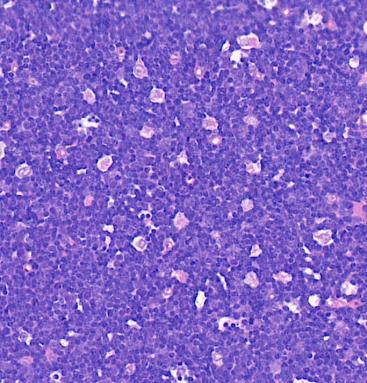

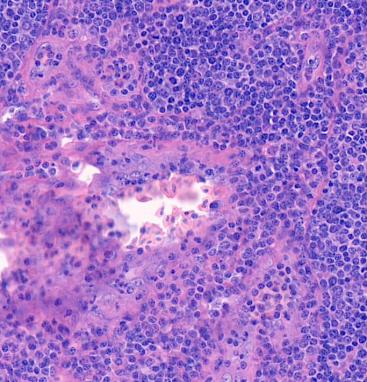

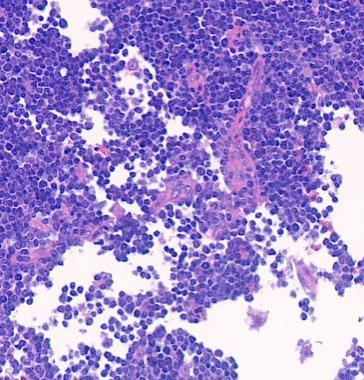

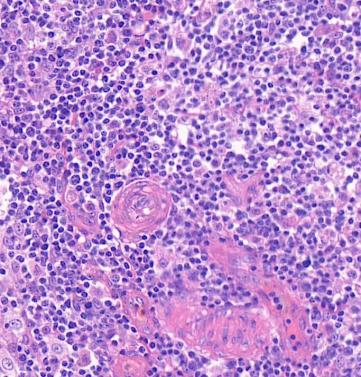

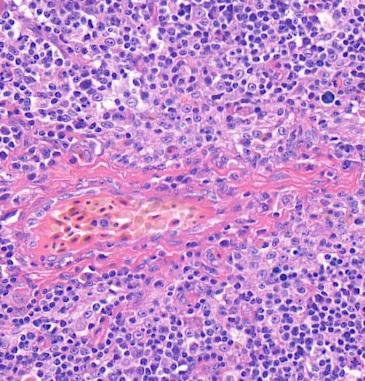


b


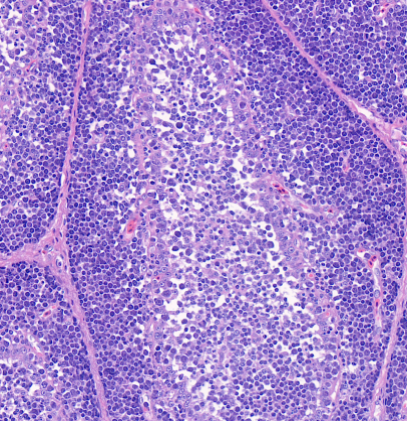

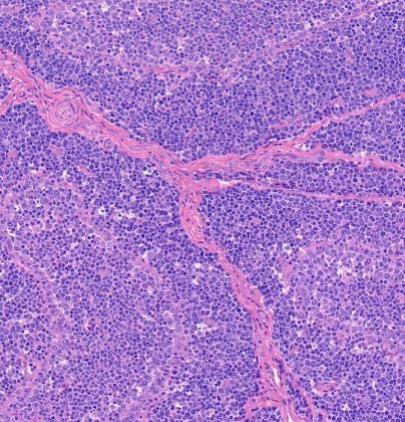

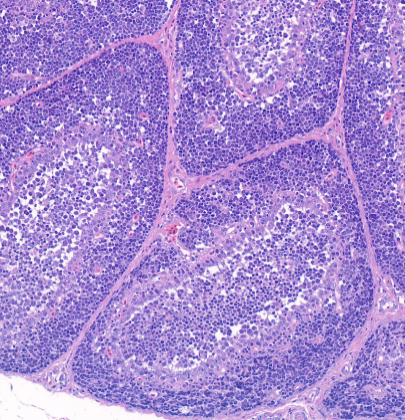


Fig.S3c.Pathological observation of duck's Bursa infected with duck distemper virus（30×）. (N). Normal control group; (V).virus infected group; (M).Drug intervention group

**Group M**

**Group N**

**Group V**

**24 h**

**36 h**

**48 h**


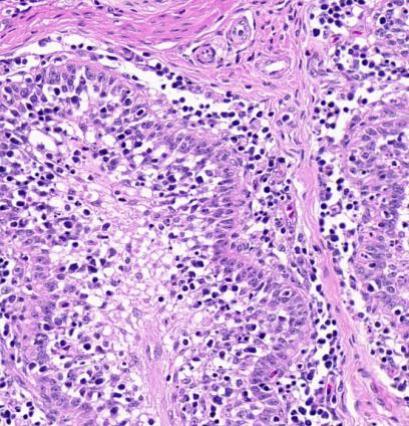

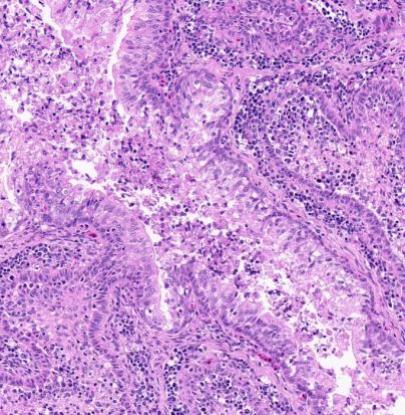

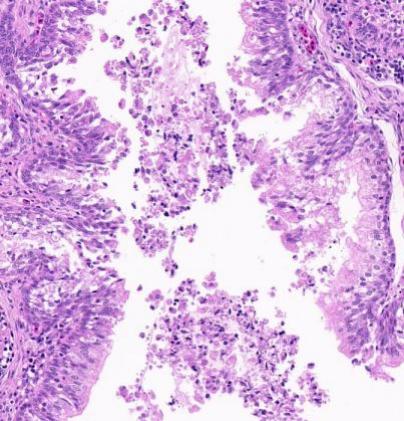

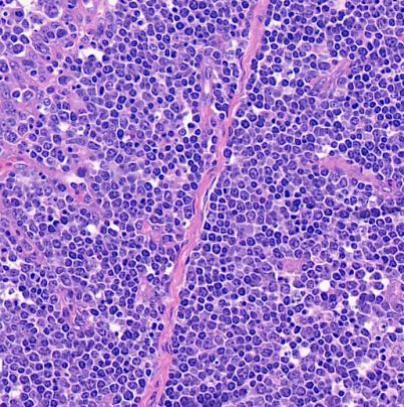

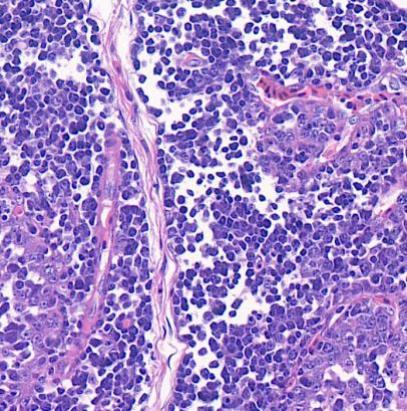

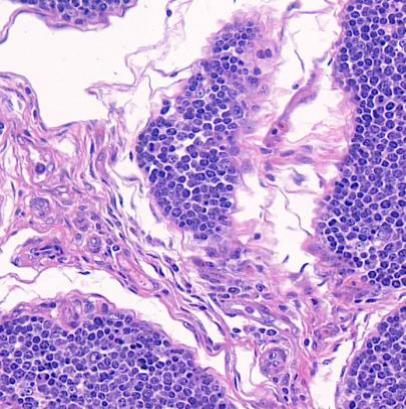


c
